# Nanoplastic Exposure at Predicted Environmental Concentrations Induces Activation of Germline Ephrin Signal Associated with Toxicity Formation in the *Caenorhabditis elegans* Offspring

**DOI:** 10.3390/toxics10110699

**Published:** 2022-11-17

**Authors:** Yue Zhao, Xin Hua, Qian Bian, Dayong Wang

**Affiliations:** 1 Key Laboratory of Environmental Medicine Engineering in Ministry of Education, Medical School, Southeast University, Nanjing 210009, China; 2 Institute of Toxicology and Risk Assessment, Jiangsu Provincial Center for Disease Control and Prevention, Nanjing 210009, China; 3 Shenzhen Ruipuxun Academy for Stem Cell & Regenerative Medicine, Shenzhen 518122, China

**Keywords:** nanoplastic, germline ephrin signal, toxicity, offspring, *C. elegans*

## Abstract

In nematode *Caenorhabditis elegans*, exposure to polystyrene nanoparticles (PS-NPs) at predicted environmental concentrations can cause induction of transgenerational toxicity. However, the underlying mechanisms for toxicity formation of PS-NP in the offspring remain largely unknown. In this study, based on high-throughput sequencing, Ephrin ligand EFN-3 was identified as a target of KSR-1/2 (two kinase suppressors of Ras) in the germline during the control of transgenerational PS-NP toxicity. At parental generation (P0-G), exposure to 0.1–10 μg/L PS-NP caused the increase in expression of germline *efn-3*, and this increase in germline *efn-3* expression could be further detected in the offspring, such as F1-G and F2-G. Germline RNAi of *efn-3* caused a resistance to transgenerational PS-NP toxicity, suggesting that the activation of germline EFN-3 at P0-G mediated transgenerational PS-NP toxicity. In the offspring, Ephrin receptor VAB-1 was further activated by the increased EFN-3 caused by PS-NP exposure at P0-G, and RNAi of *vab-1* also resulted in resistance to transgenerational PS-NP toxicity. VAB-1 acted in both the neurons and the germline to control toxicity of PS-NP in the offspring. In the neurons, VAB-1 regulated PS-NP toxicity by suppressing expressions of DBL-1, JNK-1, MPK-1, and GLB-10. In the germline, VAB-1 regulated PS-NP toxicity by increasing NDK-1 and LIN-23 expressions and decreasing EGL-1 expression. Therefore, germline Ephrin ligand EFN-3 and its receptor VAB-1 acted together to mediate the formation of transgenerational PS-NP toxicity. Our data highlight the important role of activation in germline Ephrin signals in mediating transgenerational toxicity of nanoplastics at predicted environmental concentrations in organisms.

## 1. Introduction

Over the past two decades, the usage of plastic products has increased drastically, which has led to the occurrence of plastic pollution in the amount of millions of tons per year [1]. The sources of plastic waste are associated with product wear, management policy, and human consumption. In the environment, microplastics and nanoplastics can be formed from plastic waste via weathering degradation, oxidative degradation, and biological degradation by microorganisms [2,3]. Nanoplastics are those plastic particles with sizes less than 100 nm, derived from bulk plastics through degradation or direct environmental release [4,5]. Largely due to their very small size, nanoplastic particles have the potential to easily cross some biological barriers to target different tissues in organisms [6]. The polystyrene nanoparticle (PS-NP) is a commonly examined nanoplastic. Exposure to nanoplastics, such as PS-NP, can result in different aspects of toxicity in organisms, such as embryonic toxicity, neurotoxicity, immunotoxicity, and metabolic dysfunction [7,8,9]. In addition, due to high hydrophobicity, PS-NPs exhibit strong affinity towards other pollutants and even enhance the toxicity of other pollutants such as microcystin [10]. After exposure, nanoplastics can not only be accumulated in the exposed animals (such as fish), but can also pass into the body of their next generations [11].

*Caenorhabditis elegans* is a model animal with small size, short lifespan, and high reproductive capacity. *C. elegans* is highly sensitive to toxicity of low doses of pollutants after long-term exposure [12,13,14]. Using *C. elegans* as an experimental animal, the toxic effects on reproduction, locomotion, metabolism, and development have been observed after nanoplastic exposure [15,16,17]. Due to its short life-cycle, *C. elegans* is an important model for the study of transgenerational toxicology [18,19]. It has been suggested that the predicted environmental concentrations of nanoplastics are less than 10 μg/L [20]. Exposure to nanoplastics (such as PS-NP) at predicted environmental concentrations could further cause toxicity in the offspring of nematodes [21,22], which was dependent on both size and surface modifications [23,24]. In nematodes, this induction of transgenerational nanoplastic toxicity is under the control of certain epigenetic molecular regulations. For example, transgenerational PS-NP toxicity on locomotion and reproduction is associated with alterations in some histone methyltransferases, such as MET-2 and SET-2 [25,26].

microRNA (miRNA) regulation is another form of epigenetic control of gene expression in organisms. The miRNAs with 19–22 nucleotides negatively control the expression of targeted genes by binding to their 3’-UTR [27]. More recently, we found that the decrease in *mir-38* in the germline was associated with induction of transgenerational PS-NP toxicity in nematodes [28]. During the control of transgenerational toxicity, *mir-38* activated kinase suppressors of Ras (KSR-1/2) by inhibiting its target NDK-1 in the germline in the parental generation (P0-G) [28]. However, the underlying mechanisms for the role of the decrease in germline KSR-1/2 at P0-G in mediating induction of transgenerational PS-NP toxicity remain largely unclear.

In organisms, the Ephrin signal is involved in the control of various stresses, such as water avoidance, arterial shear stress, and endoplasmic reticulum stress [29,30,31]. In nematodes, EFN-3 is the Ephrin ligand, and VAB-1 is the Ephrin receptor. In this study, based on high throughput sequencing and functional analysis, Ephrin ligand EFN-3 was identified as the target of germline KSR-1/2 at P0-G after PS-NP exposure. Moreover, the germline EFN-3 regulated the transgenerational toxicity of PS-NP at predicted environmental concentrations by activating its receptor VAB-1 in the offspring. Our results provide an important basis for the association between transgenerational PS-NP toxicity induction and germline Ephrin-mediated molecular regulation.

## 2. Materials and Methods

### 2.1. PS-NP Properties

The 20 nm PS-NPs were purchased from Janus New-Materials Co. (Nanjing, China). The analysis of transmission electron microscopy (TEM, JEOL Ltd., Tokyo, Japan) indicated the spherical morphology of PS-NPs (Figure S1). The size of the PS-NPs was 20.55 ± 3.1 nm, and the zeta potential of PS-NPs was −5.298 ± 0.697 mV based on dynamic light scattering (DLS) assay. The FTIR spectrum and Raman spectrum of PS-NPs have been reported previously [23,28].

### 2.2. C. elegans Maintenance

Information for used *C. elegans* strains is provided in Table S1. Strain maintenance was performed as previously described [32].

To perform PS-NP exposure, L1-larvae population needed to be prepared. For this aim, gravid hermaphrodite nematodes were treated with lysis solution containing 0.45 M NaOH and 2% HOCl. After release of eggs from the body, the collected eggs were transferred onto the surface of new NGM plates containing *Escherichia coli* OP50 as the food source. The eggs were allowed to develop into the synchronized L1-larvae.

### 2.3. Exposure

To examine the transgenerational PS-NP toxicity, the nematodes were exposed to PS-NP suspensions at a concentration of 0.1–10 μg/L from L1-larvae to adult day-3 (approximately for 6-day) at P0-G [33]. The PS-NP suspensions were refreshed daily throughout the exposure process. In the PS-NP suspensions, *E. coli* OP50 was added to the final concentration of ~4 × 10^6^ colony-forming unit (CFU). Before the exposure, sonication was conducted for PS-NP suspensions for 30 min at 40 kHz and 100 W. From the first filial generation (F1-G), the nematodes were allowed to develop on normal NGM plates fed with *E. coli* OP50 and without PS-NP exposure.

### 2.4. Endpoints

Both inhibition in locomotion behavior and suppression in reproductive capacity could be observed in the offspring of PS-NPs exposed nematodes [23]. Thus, locomotion behavior reflected by body bend and head thrash and brood size were used as endpoints for assessment of transgenerational PS-NPs toxicity. The frequencies of body bend and head thrash were counted as the changes of direction for bending at mid-body and posterior bulb (*y*-axis), respectively, if we considered the direction of swimming for nematodes as *x*-axis [34]. Brood size was measured as the total number of offspring produced beyond the egg stage [35]. For each exposure, 50 nematodes were examined for assay of locomotion, and 30 nematodes were examined for assay of brood size.

### 2.5. Transcriptional Expression Analysis

The reagent Trizol was used to exact total RNA of adult nematodes. The obtained RNA was assessed for quality using a Nanodrop One based on OD260/280 ratio, and then used for cDNA synthesis using a Gradient MasterCycler (Eppendorf, Hamburg, Germany). In the SYBR Green master mix for real-time polymerase chain reaction (RT-PCR), alterations in gene expression were analyzed using a StepOnePlus real-time PCR instrument. The comparative Ct method was used for quantifying the gene expression after normalization with expression of reference gene *tba-1*. To analyze the expression of certain genes in the gonad, we isolated the intact gonad. Thirty gonads were used for each treatment. Three replicates were performed. Table S2 shows the related primer information.

### 2.6. RNA Interference (RNAi)

To inhibit gene expression, RNAi knockdown was performed by feeding L1-larvae with *E. coli* HT115 expressing dsRNA of certain genes [36]. Progeny of nematodes on RNAi plates were used for PS-NP exposure. Nematodes fed with HT115 expressing empty vector of L4440 were used as control [37]. RNAi efficiency was determined by qRT-PCR (Figures S2 and S3). RNAi efficiency of *ksr-1* and *ksr-2* in the germline was reported previously [28]. To perform the tissue-specific RNAi knockdown of genes, strains of DCL569 and TU3401 were used as germline and neuronal RNAi knockdown tools, respectively (Table S1) [28].

### 2.7. HiSeq 2000 Sequencing

Previous studies have indicated that exposure to 1 μg/L PS-NP could result in transgenerational toxicity [23]. Thus, 1 μg/L was selected as the exposure concentration for PS-NP exposure. HiSeq 2000 sequencing was used to determine dysregulated genes caused by germline RNAi knockdown of *ksr-1* or *ksr-2* after exposure to PS-NP (1 μg/L). Three groups of samples were prepared: PS-NP exposed DCL-569(L4440), PS-NP exposed *ksr-1(RNAi),* and PS-NP exposed *ksr-2(RNAi)*.

Using RNAs isolated from these three groups of samples, mRNA libraries were prepared for Illumina HiSeqTM 2000 sequencing. Using Fast QC, the quality of reads was examined. Dysregulation of genes was assessed by fold change analysis and statistical significance.

### 2.8. Construct Generation and Transgene

To determine the interaction of EFN-3 and VAB-1, P*mex-5-efn-3* was constructed. The cDNA of *efn-3* was subcloned into pPD95_77 with P*mex-5* promoter to obtain the P*mex-5-efn-3*. The transgene was performed by the co-injection of constructs (50 μg/mL) and marker construct (P*dop-1::rfp*, 50 μg/mL) in the gonad. After cultivation for 3–4 days, transgenic nematodes were picked out and selected on new NGM plates for related experiments. Primers for construction generation are shown in Table S3.

### 2.9. Data Analysis

Statistical tests were performed with SPSS v19.0 software. The significant differences among treatment groups were determined by one-way or two-way ANOVA (for multi-factor comparison) followed by post-hoc test. A *p*-value of <0.01 (**) was deemed statistically significant. Statistical significance between curves for transgenerational analysis was determined by Kaplan–Meier survival analysis, followed by the log-rank test.

## 3. Results

### 3.1. Dysregulated Genes Required for Stress Response Control Were Not Candidate Targets of KSR-1/2 in Controlling Transgenerational PS-NP Toxicity

To determine the underlying mechanisms for germline KSR-1 and KSR-2 in controlling transgenerational PS-NP toxicity, we performed high throughput HiSeq 2000 sequencing on DCL569, *ksr-1(RNAi)*, and *ksr-2(RNAi)* nematodes after exposure to PS-NP (1 μg/L). In *C. elegans*, some *clec* and *lys* genes are required for stress response and innate immunity by acting as antimicrobial genes [38,39]. After PS-NP exposure, germline RNAi of *ksr-1* or *ksr-2* dysregulated the expression of some *clec* genes, including *clec-222*, *clec-266*, *clec-147*, *clec-175*, *clec-78*, *clec-87*, *clec-88*, and *clec-91* (Tables S4 and S5). However, germline RNAi of *clec-222*, *clec-266*, *clec-147*, *clec-175*, *clec-78*, *clec-87*, *clec-88*, and *clec-91* did not affect transgenerational PS-NP toxicity (Figure S4).

After PS-NP exposure, *ksr-1* or *ksr-2* knockout also dysregulated the expression of some *lys* genes, including *lys-10*, *lys-7*, and *lys-4* (Tables S4 and S5). Nevertheless, germline RNAi of *lys-10*, *lys-7*, and *lys-4* also did not influence transgenerational PS-NP toxicity (Figure S4).

After PS-NP exposure, *ksr-2* knockout further dysregulated expressions of *daf-18* and *sod-3* in insulin signaling pathway (Table S5). It has been reported that the signaling cascade from *daf-2* encoding insulin receptor to *sod-3* encoding target of DAF-16 transcriptional factor in the insulin signaling pathway does not function in the germline to control the toxicity of nanoplastics [40].

### 3.2. Identification of Ephrin Ligand EFN-3 as Candidate Downstream Target of KSR-1/2 in Controlling Transgenerational PS-NP Toxicity

**Figure 1:**
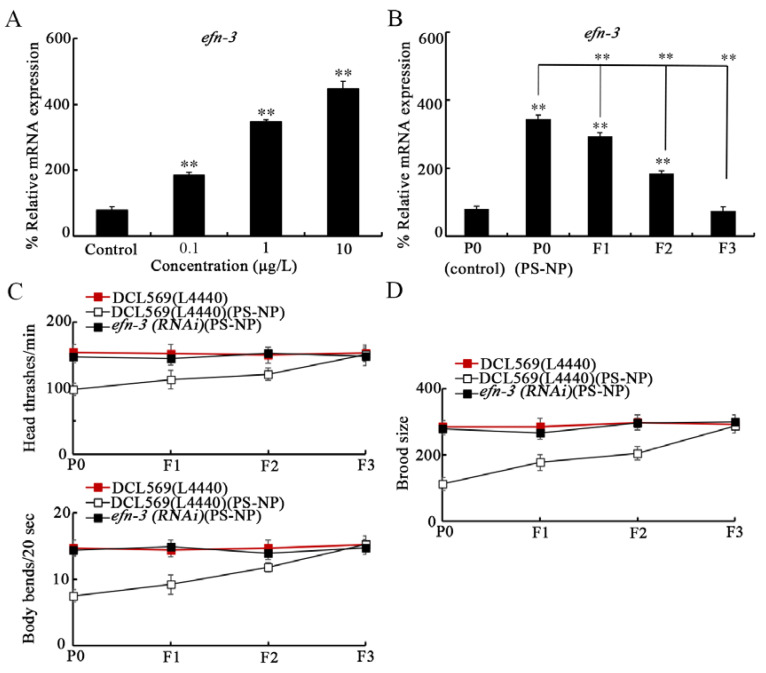
Requirement of germline Ephrin ligand EFN-3 in controlling transgenerational PS-NP toxicity. (**A**) Effect of PS-NP exposure on germline *efn-3* expression. (**B**) Transgenerational expressions of germline *efn-3* after PS-NP (1 μg/L) exposure. (**C**,**D**) Effect of germline RNAi of *efn-3* on transgenerational toxicity of PS-NP (1 μg/L) in decreasing locomotion and in inhibiting brood size. Curves of DCL569 (L4440) (PS-NP) showed a significant difference (*p* < 0.01) compared to DCL569 (L4440). After the PS-NP exposure, the curves of *efn-3 (RNAi)* showed a significant difference (*p* < 0.01) compared to DCL569 (L4440). A total of 30 intact gonads were used for the qRT-PCR assay. ** *p* < 0.01 vs. control or P0 (control) (if not specifically indicated).

Besides some dysregulated stress-response-related genes indicated above, we further found that both *ksr-1* RNAi and *ksr-2* RNAi could increase the expression of *efn-3* encoding an Ephrin ligand in 1 μg/L PS-NP exposed nematodes (Tables S3 and S4). After 0.1–10 μg/L PS-NP exposure, the germline *efn-3* expression was further increased (Figure 1A). In addition, after 1 μg/L PS-NP exposure, this increase in germline *efn-3* expression could be observed at F1-G and F2-G (Figure 1B).

Using locomotion and brood size as the endpoints, germline RNAi of *efn-3* conferred a resistance to the transgenerational PS-NP toxicity (Figure 1C,D). These observations suggested the requirement of germline Ephrin ligand EFN-3 in modulating transgenerational PS-NP toxicity.

### 3.3. Genetic Interaction between EFN-3 and KSR-1/2 in Controlling Transgenerational PS-NP Toxicity

**Figure 2:**
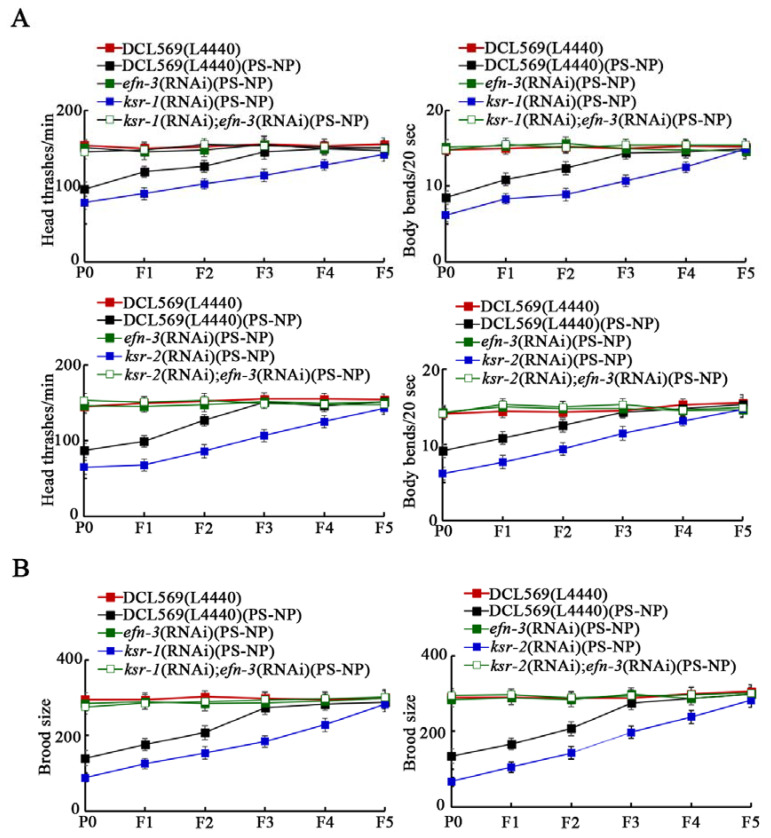
Genetic interaction between EFN-3 and KSR-1/2 in controlling transgenerational PS-NP toxicity in decreasing locomotion (**A**) and in reducing brood size (**B**). Curves of DCL569(L4440)(PS-NP) showed a significant difference (*p* < 0.01) compared to DCL569(L4440). After the PS-NP exposure, the curves of *efn-3(RNAi)*, *ksr-1(RNAi)*, and *ksr-2(RNAi)* showed a significant difference (*p* < 0.01) compared to DCL569(L4440). After the PS-NP exposure, the curves of *ksr-1(RNAi)efn-3(RNAi)* showed a significant difference (*p* < 0.01) compared to *ksr-1(RNAi)*, and the curves of *ksr-2(RNAi);efn-3(RNAi)* showed a significant difference (*p* < 0.01) compared to *ksr-2(RNAi)*.

Using locomotion behavior and brood size as the endpoints, after PS-NP toxicity, we observed resistance to transgenerational toxicity in *efn-3(RNAi)* nematodes, and susceptibility to transgenerational toxicity in *ksr-1(RNAi)* and *ksr-2(RNAi)* nematodes (Figure 2). Moreover, the susceptibility of *ksr-1(RNAi)* and *ksr-2(RNAi)* nematodes to transgenerational toxicity of PS-NP could be suppressed by germline RNAi of *efn-3*, and the *ksr-1(RNAi)efn-3(RNAi)* and *ksr-2(RNAi);efn-3(RNAi)* exhibited a resistance to transgenerational toxicity (Figure 2). Therefore, EFN-3 acted downstream of germline KSR-1/2 to regulate the transgenerational toxicity of PS-NP.

### 3.4. Ephrin Receptor VAB-1 Was Involved in Controlling Transgenerational PS-NP Toxicity

**Figure 3:**
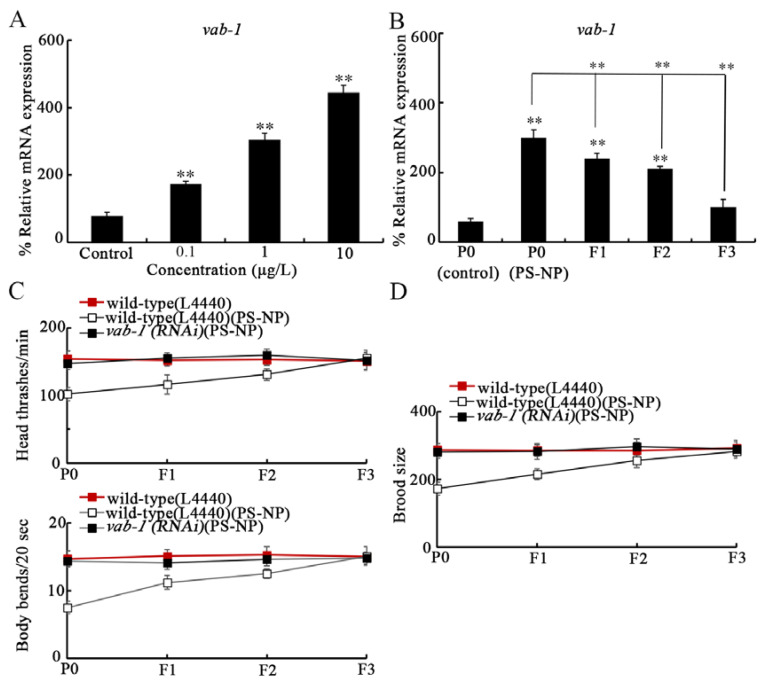
Involvement of VAB-1 in controlling transgenerational PS-NP toxicity. (**A**) Effect of PS-NP exposure on *vab-1* expression. (**B**) Transgenerational expressions of *vab-1* after PS-NP (1 μg/L) exposure. (**C**,**D**) Effect of RNAi of *vab-1* on transgenerational PS-NP toxicity in decreasing locomotion and in inhibiting brood size. Curves of wild-type(L4440)(PS-NP) showed a significant difference (*p* < 0.01) compared to wild-type(L4440). After the PS-NP exposure, the curves of *vab-1(RNAi)* showed a significant difference (*p* < 0.01) compared to wild-type(L4440). ** *p* < 0.01 vs. control or P0(control) (if not specifically indicated).

In *C. elegans*, Ephrin ligands bind to Eph receptor (EphR) tyrosine kinase VAB-1 [41]. Exposure to 0.1–10 μg/L PS-NPs increased the *vab-1* expression (Figure 3A). In addition, the increasing tendency of *vab-1* expression was observed to F2-G after 1 μg/L PS-NP exposure (Figure 3B). With locomotion behavior and brood size as endpoints, the resistance to transgenerational PS-NP toxicity was detected in *vab-1(RNAi)* nematodes (Figure 3C,D). Therefore, both Ephrin/EFN-3 and EphR/VAB-1 were involved in controlling transgenerational PS-NP toxicity.

### 3.5. Transgenerational Association between Germline Ephrin Ligand EFN-3 and Its Receptor in Controlling PS-NP Toxicity

**Figure 4:**
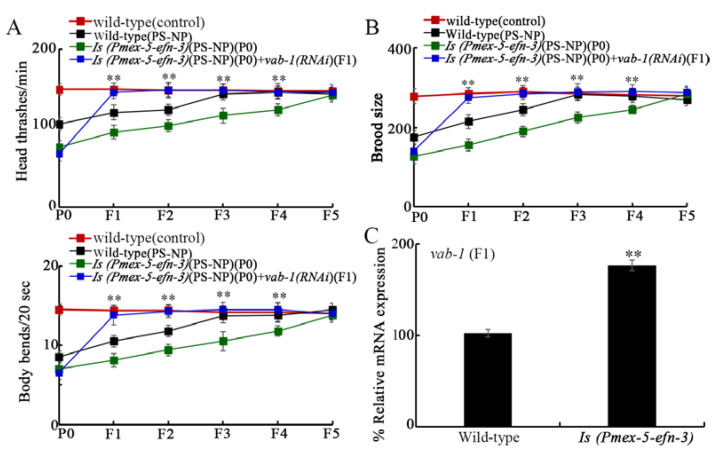
Ephrin ligand EFN-3 controlled transgenerational PS-NP toxicity by affecting function of its receptor VAB-1 transgenerationally. (**A**,**B**) Effect of *vab-1* RNAi at F1 on locomotion and brood size in 1 μg/L PS-NP exposed *Is*(P*mex-5-efn-3*) nematodes. Curves of wild-type(PS-NP) showed a significant difference (*p* < 0.01) compared to wild-type(control). Curves of *Is*(P*mex-5-efn-3*)(PS-NP)(P0) showed a significant difference (*p* < 0.01) compared to wild-type(PS-NP). ** *p* < 0.01 *vs Is*(P*mex-5-efn-3*)(PS-NP)(P0). (**C**) Effect of germline EFN-3 overexpression at P0-G on *vab-1* expression at F1-G after PS-NP (1 μg/L) exposure. ** *p* < 0.01 vs. wild-type.

To determine the interaction between germline Ephrin ligand EFN-3 and its receptor VAB-1 in controlling transgenerational PS-NP toxicity, transgenic strain *Is(Pmex-5-efn-3)* overexpressing germline EFN-3 was obtained. In these *Is(Pmex-5-efn-3)* nematodes, the susceptibility to transgenerational PS-NP toxicity on locomotion and reproduction was observed (Figure 4A,B). After exposure of *Is(Pmex-5-efn-3)* nematodes to PS-NP at P0-G, RNAi of *vab-1* at F1-G noticeably suppressed the formation of susceptibility to PS-NP toxicity observed in *Is(Pmex-5-efn-3)* nematodes from F1-G to F4-G (Figure 4A,B). Moreover, after PS-NP exposure, germline overexpression of EFN-3 significantly increased expression of *vab-1* at the F1-G (Figure 4C). These observations suggest the potential transgenerational association between germline EFN-3 and its receptor VAB-1 in controlling PS-NP toxicity induction in the offspring.

### 3.6. Tissue-Specific Activity of VAB-1 in Controlling Transgenerational PS-NPs Toxicity

**Figure 5:**
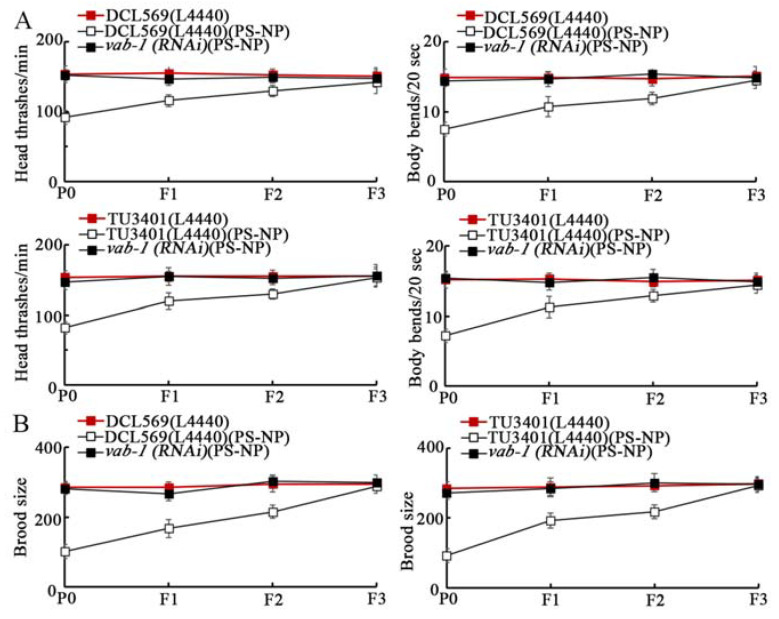
Tissue-specific activity of VAB-1 in controlling transgenerational toxicity of PS-NP (1 μg/L) in decreasing locomotion (**A**) and in reducing brood size (**B**). Curves of DCL569(L4440)(PS-NP) showed a significant difference (*p* < 0.01) compared to DCL569(L4440). Curves of TU3401(L4440)(PS-NP) showed a significant difference (*p* < 0.01) compared to TU341(L4440). After PS-NP exposure, the curves of *vab-1(RNAi)* showed a significant difference (*p* < 0.01) compared to DCL569(L4440) or TU3401(L4440).

In nematodes, VAB-1 can be expressed in neurons and reproductive tissues [42,43]. Both germline and neuronal RNAi of *vab-1* caused the resistance to transgenerational PS-NP toxicity as reflected by the endpoints of locomotion behavior and brood size (Figure 5A,B). That is, VAB-1 could function in both the germline and neurons to regulate the toxicity induction of PS-NPs in the offspring.

### 3.7. Identification of Potential Targets of Neuronal VAB-1 in Controlling PS-NP Toxicity

**Figure 6:**
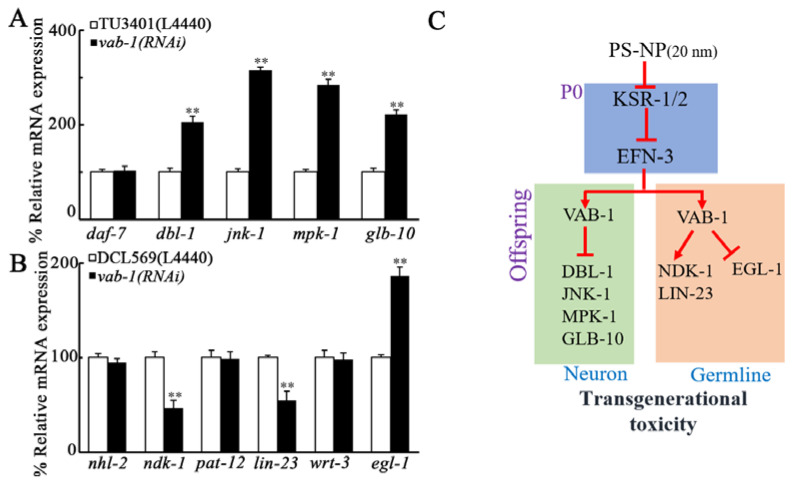
Identification of downstream targets of neuronal and germline VAB-1 in controlling PS-NP toxicity. (**A**) Effect of neuronal *vab-1* RNAi on gene expression in 1 μg/L PS-NP-exposed nematodes. (**B**) Effect of germline *vab-1* RNAi on gene expression in 1 μg/L PS-NP-exposed nematodes. ** *p* < 0.01 vs. TU3401(L4440) or DCL569(L4440). (**C**) A diagram showing the molecular basis for germline Ephrin signal in controlling transgenerational PS-NP toxicity in nematodes.

In the neurons, some molecular signals have been proven to be involved in the control of PS-NP toxicity [44,45,46,47,48]. Among these signals, DAF-7 and DBL-1 are two TGF-β ligands, JNK-1 is JNK MAPK, MPK-1 is ERK MAPK, and GLB-10 is a globin protein. In PS-NP-exposed nematodes, although *daf-7* expression was not altered by neuronal RNAi of *vab-1*, expressions of *dbl-1*, *jnk-1*, *mpk-1*, and *glb-10* were significantly increased by neuronal RNAi of *vab-1* (Figure 6A).

### 3.8. Identification of Potential Targets of Germline VAB-1 in Controlling PS-NP Toxicity

In the germline, some molecular signals were also raised to be required for controlling PS-NP toxicity [49,50]. Among these signals, EGL-1 is a BH3 protein governing germline cell death, WRT-3 is a Hedgehog ligand, LIN-23 is an E3 ubiquitin ligase, PAT-12 is a component of hemidesmosomes, NHL-2 is a miRISC cofactor, and NDK-1 is NM23-H1 homolog. In PS-NP exposed nematodes, expressions of *nhl-2*, *pat-12*, and *wrt-3* were not affected by germline RNAi of *vab-1* (Figure 6B). In contrast, after PS-NP exposure, *lin-23* and *ndk-1* expressions were significantly decreased by germline RNAi of *vab-1*, and *egl-1* expression was increased by germline RNAi of *vab-1* (Figure 6B).

## 4. Discussion

In nematodes, during the control of transgenerational toxicity of pollutants, different forms of epigenetic regulations play important functions. For example, histone methylation- or demethylation-related molecular signals are associated with transgenerational toxicity induction of different pollutants, such as arsenite, CuO nanoparticles, and nanoplastics [26,51,52]. In addition, alterations in certain long non-coding RNAs also mediate the transgenerational toxicity induction of multiwalled carbon nanotubes [35]. Recently, we further found that certain miRNAs, such as *mir-38*, are also involved in controlling transgenerational PS-NP toxicity [28]. In the germline, the *mir-38* regulated transgenerational PS-NP toxicity by inhibiting NDK1-KSR-1/2 axis. However, the underlying mechanism for how decreases in germline KSR-1/2 mediated the PS-NP toxicity induction in the offspring remains largely unclear. For this reason, we performed high-throughput sequencing for *ksr-1(RNAi)* and *ksr-2(RNAi)* nematodes after PS-NP exposure. Nevertheless, among the dysregulated stress-response-related genes caused by germline RNAi *ksr-1* and *ksr-2* after PS-NP exposure, we did not observe their function in controlling transgenerational toxicity (Figure S4). These observations implied that, although these dysregulated stress-response-related genes can be expressed in the germline, they may be not able to exert their function in regulating stress responses to PS-NP in this tissue.

For the underlying molecular mechanisms of germline KSR-1/2 in controlling transgenerational toxicity, we assumed that the KSR-1/2 may affect certain secretory ligands in the germline so as to regulate the induction of PS toxicity in the offspring. In this study, we further found that the expression of Ephrin ligand EFN-3 was increased by germline RNAi of *ksr-1* or *ksr-2* in PS-NP exposed nematodes (Tables S4 and S5). The EFN-3 expression showed a pattern of transgenerational increase after PS-NP exposure (Figure 1A,B), and the *efn-3(RNAi)* nematodes showed resistance to transgenerational PS-NP toxicity (Figure 1C,D). Moreover, in the germline, EFN-3 could function downstream of KSR-1/2 to control transgenerational PS-NP toxicity (Figure 2). These observations suggested that, during the control of transgenerational toxicity, germline *mir-38* suppressed the function of Ephrin signaling by inhibiting NDK1-KSR1/2 axis. That is, EFN-3 is one of the downstream secretory targets of KSR-1/2 in the germline. Previous studies have indicated the function of EFN-3 in the control of epidermal organization [41]. Our data here further demonstrate the function of germline EFN-3 in regulating stress responses. In nematodes, there are four Ephrin ligands (EFN-1-4). Nevertheless, EFN-1, EFN-2 and EFN-4 are not expressed in the germline (https://wormbase.org, accessed on 1 October 2022). In addition, PS-NP (0.1–10 μg/L) exposure did not affect the expression of *efn-1*, *efn-2*, and *efn-4* (data not shown).

The activation of Ephrin receptor VAB-1 by ligand EFN-3 has been confirmed by biochemical analysis [41]. In *C. elegans*, Ephrin ligands (such as EFN-1 and EFN-2) can function together with receptor VAB-1 in regulating cellular organization during development [41]. In this study, we also observed the transgenerational increase in VAB-1 expression after PS-NP exposure (Figure 3A,B), and the *vab-1(RNAi)* nematodes also exhibited resistance to transgenerational PS-NP toxicity (Figure 3C,D). Therefore, Ephrin ligand EFN-3 acted together with its receptor VAB-1 to regulate transgenerational toxicity of PS-NP in nematodes (Figure 6C).

Several lines of evidence were further raised in this study to suggest the potential involvement of transgenerational communication of Ephrin signaling in controlling transgenerational toxicity of PS-NP. On the one hand, we found that RNAi of *vab-1* encoding Ephrin receptor at F1-G could inhibit the induction of transgenerational toxicity in the PS-NP-exposed transgenic strain overexpressing germline EFN-3 (Figure 4A,B). On the other hand, in PS-NP exposed nematodes, germline overexpression of EFN-3 could increase VAB-1 expression at F1-G (Figure 4C). These observations suggested that the increase in germline Ephrin ligand EFN-3 caused by PS-NP exposure mediated the transgenerational toxicity by functioning upstream of its receptor VAB-1 in the offspring. Recently, we also found that several germline insulin ligands (INS-3, INS-39, and DAF-28) and germline Wnt ligand (LIN-44) could be activated by PS-NP exposure, which then potentially mediates the transgenerational toxicity induction by activating or inhibiting corresponding receptors in the offspring [22,53].

VAB-1 is mainly expressed in neurons and reproductive tissues [42,43]. Our tissue-specific activity analysis on VAB-1 indicated that VAB-1 acted in both neurons and germline to affect PS-NP toxicity in the offspring (Figure 6C). This implied that, after the activation in the germline at P0-G, the EFN-3 could regulate transgenerational PS-NP toxicity by further activating receptor VAB-1 in neurons and germline, respectively, in the offspring. During development, VAB-1 has been found to be required for neuronal morphogenesis, neuronal regeneration, and axon guidance [54,55,56]. In addition, VAB-1 is also involved in the control of oocyte meiotic maturation and germline apoptosis [57,58].

In this study, we identified several targets for neuronal VAB-1 in regulating PS-NP toxicity (Figure 6C). Among the examined two genes encoding TGF-β ligands, only expression of *dbl-1* was increased by neuronal RNAi of v*ab-1* after PS-NP exposure (Figure 6A). Expressions of both *jnk-1* encoding JNK MAPK and *mpk-1* encoding ERK MAPK could be increased in PS-NP-exposed *vab-1(RNAi)* nematodes (Figure 6A). In addition, in PS-NP-exposed nematodes, neuronal RNAi of *vab-1* also increased expression of *glb-10* encoding globin (Figure 6A). Therefore, in the neurons, Ephrin receptor VAB-1 potentially regulated PS-NP toxicity by suppressing functions of multiple downstream molecular signals, including TGF-β, JNK MAPK, ERK MAPK, and GLB-10 signals. Neuronal RNAi of *dbl-1*, *jnk-1*, *mpk-1*, and *glb-10* induced a susceptibility to PS-NP toxicity [44,46,47,48], which further supports the observed function of neuronal VAB-1 in controlling PS-NP toxicity in the offspring.

In the germline, we further identified LIN-23, NDK-1, and EGL-1 as the targets of Ephrin receptor VAB-1 during the control of PS-NP toxicity (Figure 6C). In PS-NP-exposed nematodes, germline RNAi of *vab-1* caused the decrease in expressions of *ndk-1* and *lin-23*, and the increase in *egl-1* expression (Figure 6B). This suggested that, in PS-NP-exposed nematodes, germline RNAi of *vab-1* induced two different downstream molecular responses. In *C. elegans*, E3 ubiquitin ligase LIN-23 exhibits the function of cell division limitation [59], which is helpful for understanding the function of Ephrin signaling pathway and the observed reduction in brood size in PS-NP exposed nematodes. In *C. elegans*, EGL-1 is required for controlling stress responses, and *egl-1* RNAi caused a susceptibility to toxicants or stresses, such as PS-NP and pathogen infection [48,60]. Moreover, we observed the increase in NDK-1 expression in PS-NP exposed nematodes with germline RNAi of *vab-1* (Figure 6B). Recently, we found that the decreased germline *mir-38* could increase KSR-1/2 expression by suppressing the function of NDK-1 [28]. This suggested that, once the germline *mir-38* was decreased by PS-NP exposure at P0-G, this could drive the suppression of NDK-1-KSR-1/2-EFN-3 signaling cascade transgenerationally. These findings are useful for explaining the observed toxicity in the offspring, such as those at both F1-G and F2-G [23].

## 5. Conclusions

Together, we here investigated the role of germline Ephrin signal in regulating transgenerational PS-NP toxicity using *C. elegans* as an animal model. Activation of Ephrin ligand EFN-3 could mediate the induction of transgenerational toxicity of PS-NP at predicted environmental concentrations by acting downstream of KSR-1/2, two kinase suppressors of Ras, in the germline. The activated germline EFN-3 by PS-NP exposure induced toxicity in the offspring by activating corresponding Ephrin receptor VAB-1 in both neurons and germline. In the neurons and the germline, VAB-1 controlled PS-NP toxicity by activating and/or inhibiting certain downstream targets. Our results are helpful for understanding how toxicity is induced in offspring (such as F1-G and F2-G) after PS-NP exposure at P0-G. Nevertheless, considering the examined *C. elegans* are hermaphrodites, the role and the underlying mechanism of germline Ephrin signals in regulating transgenerational PS-NP toxicity still need to be further elucidated in mammal models.

## Data Availability

Not applicable.

## Supplementary Materials

The following supporting information can be downloaded at: https://www.mdpi.com/article/10.3390/toxics10110699/s1, Figure S1: TEM image of PS-NPs before the sonication; Figure S2: Germline RNAi efficiency of efn-3, clec-222, clec-266, clec-147, clec-175, clec-78, clec-87, clec-88, clec-91, lys-10, lys-4, or lys-7. ** *p* < 0.01 vs. DCL569(L4440); Figure S3: RNAi efficiency of *vab-1*. ** *p* < 0.01 vs. wild-type(L4440), DCL569(L4440), or TU3401(L4440); Figure S4: Effects of germline RNAi of *clec-222*, *clec-266*, *clec-147*, *clec-175*, *clec-78*, *clec-87*, *clec-88*, *clec-91*, *lys-10*, *lys-4*, or *lys-7* on transgenerational PS-NP toxicity in decreasing locomotion behavior. Exposure concentration of PS-NP was 1 μg/L. Curves of DCL569(L4440)(PS-NP) showed a significant difference (*p* < 0.01) compared to DCL569(L4440). After the PS-NP exposure, the curve of *clec-222(RNAi)* (*p* = 0.812), *clec-266(RNAi)* (*p* = 0.879), *clec-147(RNAi)* (*p* = 0.756), *clec-175(RNAi)* (*p* = 0.857), *clec-78(RNAi)* (*p* = 0.925), *clec-87(RNAi)* (*p* = 0.970), *clec-88(RNAi)* (*p* = 0.946), *clec-91(RNAi)* (*p* = 0.879), *lys-10(RNAi)* (*p* = 0.896), *lys-4(RNAi)* (*p* = 0.948), and *lys-7(RNAi)* (*p* = 0.978), did not show significant difference compared to DCL569(L4440); Table S1: Information for *C. elegans* strains; Table S2: Primer information for qRT-PCR; Table S3: Primer information for constructs generation; Table S4: Dysregulated genes by germline RNAi knockdown of *ksr-1* after PS-NPs exposure; Table S5: Dysregulated genes by germline RNAi knockdown of *ksr-2* after PS-NPs exposure.
