# Supplementary material for: Nanoplastic Exposure at Predicted Environmental Concentrations Induces Activation of Germline Ephrin Signal Associated with Toxicity Formation in the Caenorhabditis elegans Offspring
Source: Toxics. 2022 Nov 17;10(11):699. doi: 10.3390/toxics10110699 (PMC9696181; doi:10.3390/toxics10110699)
Supplement: Supplementary file 1 [file toxics-10-00699-s001.zip › toxics-2018200-supplementary.pdf]

toxics MDPI

Supplementary Materials: Nanoplastic Exposure at Predicted Environmental Concentrations Induces Activation of Germline Ephrin Signal Associated with Toxicity Formation in the *Caenorhabditis elegans* Offspring

Yue Zhao, Xin Hua, Qian Bian and Dayong Wang

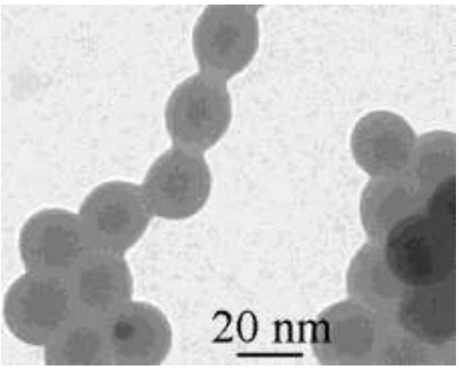

Figure S1. TEM image of PS-NPs before the sonication.

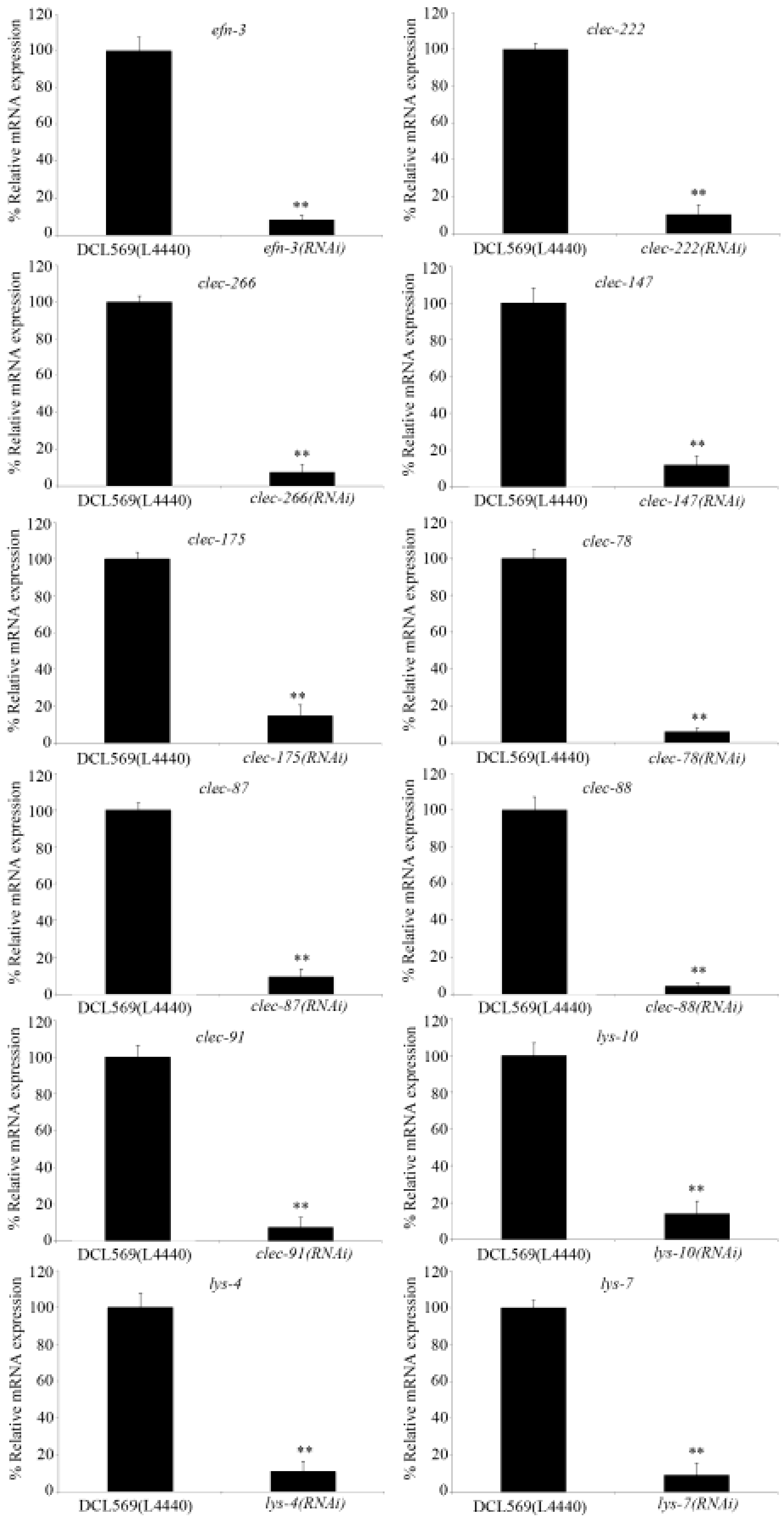

Figure S2. Germline RNAi efficiency of efn-3, clec-222, clec-266, clec-147, clec-175, clec-78, clec-87, clec-88, clec-91, lys-10, lys-4, or lys-7. **P < 0.01 vs DCL569(L4440).

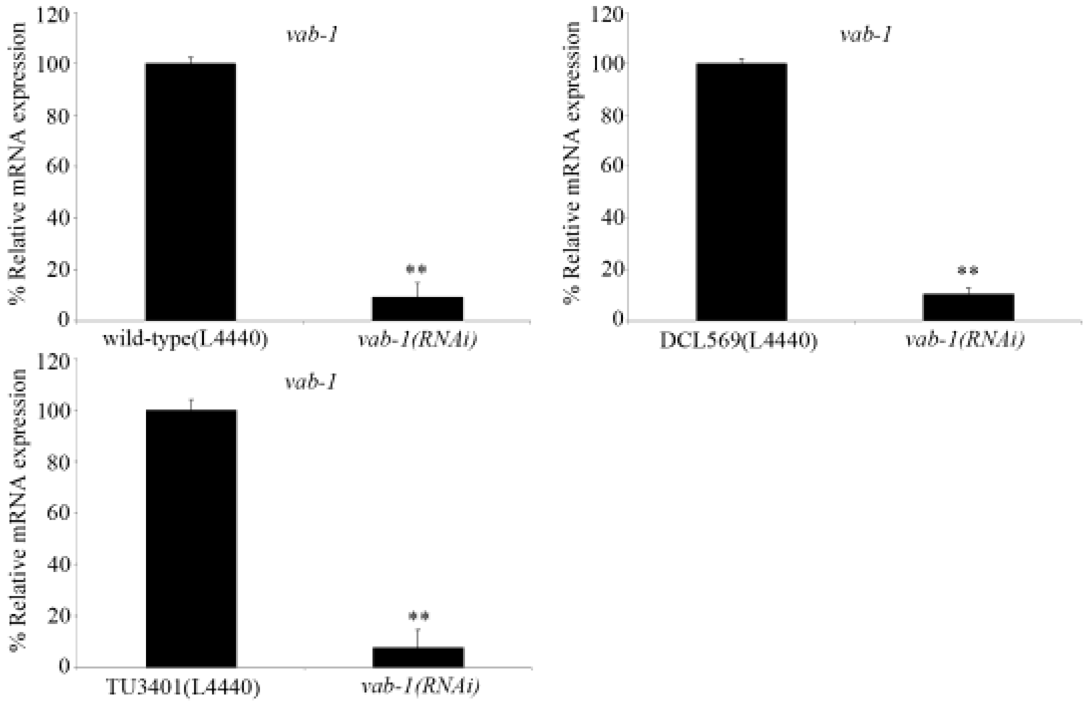

Figure S3. RNAi efficiency of *vab-1*. $^{**}P < 0.01$ *vs* wild-type(L4440), DCL569(L4440), or TU3401(L4440).

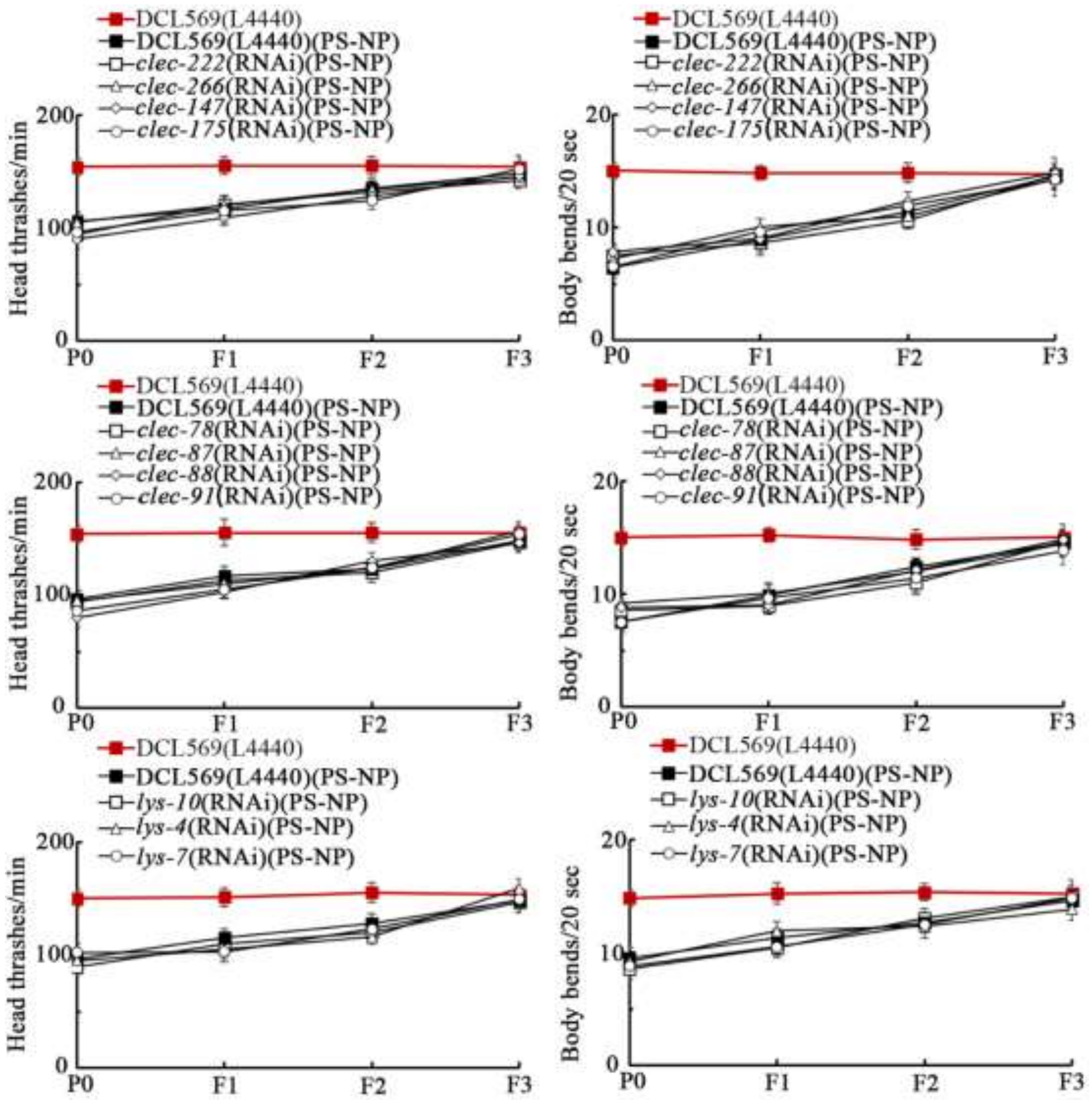

Figure S4. Effects of germline RNAi of *clec-222*, *clec-266*, *clec-147*, *clec-175*, *clec-78*, *clec-87*, *clec-88*, *clec-91*, *lys-10*, *lys-4*, or *lys-7* on transgenerational PS-NP toxicity in decreasing locomotion behavior. Exposure concentration of PS-NP was 1 μg/L. Curves of DCL569(L4440)(PS-NP) showed a significant difference ($P < 0.01$) compared to DCL569(L4440). After the PS-NP exposure, the curve of *clec-222(RNAi)* ($P = 0.812$), *clec-266(RNAi)* ($P = 0.879$), *clec-147(RNAi)* ($P = 0.756$), *clec-175(RNAi)* ($P = 0.857$), *clec-78(RNAi)* ($P = 0.925$), *clec-87(RNAi)* ($P = 0.970$), *clec-88(RNAi)* ($P = 0.946$), *clec-91(RNAi)* ($P = 0.879$), *lys-10(RNAi)* ($P = 0.896$), *lys-4(RNAi)* ($P = 0.948$), and *lys-7(RNAi)* ($P = 0.978$), did not show significant difference compared to DCL569(L4440).

Table S1. Information for *C. elegans* strains.

Strains	Genotype	Description
N2		Wild-type
DCL569	*mkcSi13*[*sun-1p::rde-1::sun-1 3′UTR* + *unc-119*(+)];*rde-1*(*mkc36*)	Germline RNAi knockdown tool
TU3401	*sid-1(pk3321); [pCFJ90(myo-2p::mCherry)punc-119p::sid-1]*	Neuronal RNAi knockdown tool
	*Is(*P*mex-5-efn-3)*	Nematodes overexpressing germline EFN-3

Table S2. Primer information for qRT-PCR.

Gene	Forward primer (5′-3′)	Reverse primer (5′-3′)
efn-3	CGGAGTTTGTGCAACCGAAA	ACCCAACTACCGAGATTGCC
vab-1	CCCGTGGTGTCAGAAGTGAA	GTGGTGGTAGTCGATAGCCG
daf-7	CCCTTCATCCCCAACAGACC	GACATTGGCGATTGAGACGC
dbl-1	TTTTGCGGCGAACAAATCGT	TTCGCTGTTGCCTGTTTGTG
jnk-1	TATGCTCCACCTCCACTT	GGGTTCTTGCGTAATCTG
mpk-1	AACGGGCATCCACAAGCG	AGTGCAGAGGCGACCATCC
glb-10	TCAACGATCCGCGGAAAGAA	GAAATCAACATGCCGGGCTC
nhl-2	GGGGACGCCCTGTTTATTCA	TGATTTGCAGCCAAGGCAAC
ndk-1	CCAAGGCCCATCTTGAGGTT	ATCCGTGGCAGATATTGCGT
pat-12	CAAACGGAGAGGTCCGTCAA	CGGCTCATGTCATCGTCGTA
lin-23	TGCCGGGGACAATGATGAAT	GTCAAAAGCAGCCATTTCTTGC
wrt-3	GCTTGGCGGATTACTGTGGA	AGCTGGTTTCACATCGGGAAT
egl-1	GCCTCAACCTCTTCGGATCT	GCACATTGCTGCTAGCTTGG
tba-1	TCAACACTGCCATCGCCGCC	TCCAAGCGAGACCAGGCTTCAG

Table S3. Primer information for constructs generation.

Gene	Forward primer (5′-3′)	Reverse primer (5′-3′)
P*mex-5*	ATACTGCAGTTGTAGGCTCTTCTTGCA	TCAGGATCCTCTCTGTCTGAAACATTC
efn-3/F15A2.5	ATAGGATCCATGAGCTCAAGCTGGGCC	CAGCTCGAGCTAAAACAAAACACCGCT

Table S4. Dysregulated genes by germline RNAi knockdown of *ksr-1* after PS-NPs exposure.

Gene name	log2FC	Expressed in germline
Y57A10B.6	8.769209	+
C08F1.10	8.422187	
Y82E9BR.17	4.506934	
T05D4.2	4.418915	
Y73C8C.3	3.852964	
vet-6	3.749902	
pes-2.1	3.557689	
C17E4.2	3.548801	+
Y106G6D.1	3.425848	
pap-2	3.37	
tbx-11	3.317655	+
Y25C1A.6	3.295668	
clec-266	3.258693	+
fbxb-84	3.236067	
C08F1.6	3.187794	
T22B2.1	3.181919	
T12D8.5	3.154467	
efn-3	3.134561	+
pes-2.2	3.112363	
fbxb-103	3.063931	
Y47H10A.2	3.033427	+
W04A8.5	2.962996	+
Y43F11A.1	2.894374	
sdz-30	2.857534	
Y44A6C.2	2.797981	
skr-12	2.752062	
sepa-1	2.705083	
sdz-37	2.672532	+
C35E7.5	2.654376	+
Y43F11A.4	2.625558	
W08E12.2	2.541129	
fbxb-15	2.452672	
stdh-2	2.424543	
K01G12.3	2.415352	+
pes-10	2.402182	
ech-9	2.393005	
dmsr-16	2.390111	
Y106G6D.2	2.385238	
Y65B4BR.1	2.351968	
skr-8	2.337743	
sdz-25	2.326385	
F17E9.2	2.285144	
M116.1	2.276742	
btb-11	2.25259	
fbxc-28	2.247712	+
fbxc-51	2.230555	
dsl-2	2.217453	+
T24C4.2	2.214628	+
clec-222	2.210811	+
btb-8	2.190256	+
btb-10	2.177559	
Y82E9BR.17	2.128212	
fkh-4	2.118582	+

skr-7	2.089803	+
F10A3.1	2.059092	
K10G6.5	2.040173	
spp-9	-4.42418	
Y39G8B.7	-2.92212	
dct-19	-2.92118	
F25D1.3	-2.82734	
C05B5.12	-2.70422	
F58B4.7	-2.52453	
oac-42	-2.45072	
grl-2	-2.33915	
F40C5.2	-2.33238	+
nhr-290	-2.31174	
lbp-8	-2.25486	
C35E7.5	-2.20382	
F41G3.3	-2.1741	
F36H2.4	-2.1353	
nhr-175	-2.13115	
R01E6.5	-2.11718	
ugt-34	-2.10559	
ZK945.8	-2.0919	
ZK402.5	-2.07473	+
ifa-3	-2.0724	
T05B4.12	-2.05264	
F31B9.4	-2.03284	
C05E11.7	-2.01693	
F49D11.14	-2.01086	

Note:.
-Expression information of candidate genes is from https://wormbase.org.
-Statistical significance: $P < 0.01$.

Table S5. Dysregulated genes by germline RNAi knockdown of *ksr-2* after PS-NPs exposure.

Gene name	log2FC	Expressed in germ line
C29F5.8	14.4158093432	
C41G6.13	14.2060317226	
Y75B12B.8	14.1977543808	
F47B8.14	13.6746335916	
F47B8.18	13.4128136637	
clec-19	13.3221629302	
Y54G2A.57	13.2858080250	
K04A8.21	13.2723575788	
F57F4.2	13.1769287341	
oac-39	13.1563293514	
scl-1	12.4149876014	
ZK643.6	12.3524449265	
F17B5.4	12.0182831028	
M6.11	12.0129012063	
oac-41	12.0129012063	
ZK285.2	11.9210726265	+
F48G7.7	11.7697491878	
wrt-7	11.6638472222	
K08D12.7	11.5458293751	
C43F9.4	11.3926371915	
Y59A8B.26	11.2851255821	
Y59A8B.19	11.2024347777	
C03G6.6	11.1689522466	
F20E11.17	11.1096813532	
K09D9.9	11.0531239394	
T04C12.1	11.0319951941	
C33B4.5	10.9666863624	
Y71F9AM.8	10.9328887457	
ttr-19	10.9041063600	
mltn-7	10.8865572072	
C13A2.6	10.8447594452	
F40G12.5	10.7508943479	
ilys-6	10.7379045839	
C13A2.7	10.7049090816	
F46H5.11	10.6642917918	
Y57E12B.11	10.6435461157	
lipl-8	10.6011377708	
ilys-3	10.4815478651	
R08E5.4	10.4815478651	
sek-5	10.3931736897	
Y60C6A.2	10.3339794532	
F39G3.4	10.2812336928	
T20G5.13	10.2541195756	
F07G11.3	10.2077652757	
C07G3.10	10.1887983301	+
C54F6.17	10.1792205217	
C41C4.9	10.1695787026	
C54F6.15	10.1203786774	+
K01A2.9	10.1203786774	
cnc-9	10.0485519812	
Y97E10B.1	9.9506156788	
K04A8.19	9.9164349537	
srj-32	9.8695632574	

Y9C9A.5	9.8333822774	
Y71H9A.10	9.8333822774	
spp-20	9.8211174635	
C44H9.7	9.8211174635	
clec-38	9.7709881605	
Y57A10B.2	9.6923687753	
gmd-2	9.6923687753	
B0554.1	9.6513918062	
W02D7.11	9.6513918062	
Y39H10A.1	9.6374700324	
C13A2.9	9.6130456156	
fbxa-142	9.6092168562	
grd-8	9.5657717631	
scl-12	9.5502068167	
C18H7.1	9.5358124185	
ugt-27	9.4903230740	
F15H10.12	9.4903230740	
T05A8.6	9.3942460880	
clec-248	9.3264453450	
fip-1	9.2913114010	
scl-11	9.2363192623	
amt-1	9.1995403879	
pals-29	9.1804642818	
col-51	9.1570853953	
W07G4.7	9.1216741910	
K04A8.18	9.1015327855	
ptr-9	9.1015327855	
smf-2	9.1010563993	
F56H9.8	9.0393643677	
F58E6.4	8.9963787319	
F57A8.7	8.9743958841	
otpl-4	8.9293990462	
F01G10.4	8.9293990462	
Y46E12A.5	8.8591576650	
K07A1.20	8.8103551889	
K01A2.12	8.8103551889	
R13D11.10	8.8103551889	
T05H10.3	8.7928646897	+
col-37	8.7657156607	
F07C4.10	8.7598438831	
F15H10.9	8.7598438831	
T28A11.19	8.7598438831	
col-2	8.7514506370	
col-183	8.7435447567	
ceh-76	8.7339091846	
C13A2.10	8.7074997216	
col-185	8.6936995865	
col-108	8.6734093412	
col-85	8.6595841383	
Y68A4A.10	8.6531846656	
chil-15	8.6531846656	
C17G10.10	8.6531846656	
F56F11.1	8.6252405523	
str-143	8.6252405523	
C13A2.12	8.6010077679	
zmp-5	8.5967444698	

col-52	8.5967444698	
W09C2.9	8.5967444698	
clec-101	8.5648831520	
W08E12.9	8.5380060416	
F35F10.7	8.5077149713	
srj-23	8.4767742398	
scl-13	8.4721248429	
C03H5.4	8.4451553682	
T13C5.10	8.4451553682	
col-158	8.4249954220	
col-40	8.4160908992	
grl-25	8.3679435992	
grl-23	8.3623407952	
cut-1	8.3489668420	
F15H10.5	8.3475681220	
pals-6	8.3264537385	
col-50	8.2968940360	
col-102	8.2354274728	
col-36	8.1770731522	
col-72	8.1758948170	
F47B8.13	8.0862438300	
grl-9	8.0049480838	
F07G11.1	7.9920689899	
grl-20	7.9793010676	
col-43	7.9427192380	
col-84	7.9362265688	
col-44	7.8208344554	
cri-1	7.8041693525	
asp-9	7.7249224148	
col-45	7.7155579688	
daf-42	7.6092721083	
rocf-1	7.5042968541	
spp-11	7.4466157304	
col-35	7.4378905490	
K01D12.10	7.4235434897	
C33G8.13	7.4109644501	
Y57E12B.4	7.3693907473	
C10F3.7	7.2915223058	
clec-59	7.2822627174	
C08A9.3	7.2092000018	+
col-114	7.0790054940	
ZK355.8	7.0584232676	
grd-7	7.0562802925	+
fbxa-165	6.9925941112	
col-164	6.9741703436	
C13A2.5	6.9590233001	
ZK355.3	6.8818952618	
col-123	6.8277538199	
col-163	6.8165929482	
hsp-12.6	6.7998048515	
grl-19	6.7868862221	
F23F1.2	6.7074946380	
grl-24	6.6893526528	
R05A10.1	6.6514327805	
F32G8.2	6.6359057667	
F53F4.4	6.5905106649	

Y6E2A.4	6.5882426273	
Y71F9AR.10	6.5541241402	
Y60C6A.3	6.4782968562	
H39E23.3	6.4619670867	
tyr-3	6.3933339477	
grl-17	6.3781746585	
catp-2	6.3421178700	
droe-8	6.2915945048	
C13A2.4	6.2631016547	
C13A2.3	6.1370885731	
C46A5.4	6.0676370680	
acs-2	6.0415850302	
K08D9.2	6.0401174322	
cyp-33B1	6.0335143122	
W01C9.2	5.9936130396	
H14N18.2	5.9832626873	
grd-9	5.9637987310	
K09E2.1	5.9626432555	
K08D9.6	5.9248473295	
cpr-2	5.8599826806	
nas-10	5.8307828215	
pals-32	5.7509061380	
grd-4	5.7216210449	
dmd-10	5.7195741932	
nhr-246	5.6817016154	
Y57E12B.1	5.6736163529	
nas-28	5.6729154236	
W07B8.4	5.6427805764	
F36G9.7	5.5959710902	
Y46H3A.5	5.5686571552	
F53F4.7	5.5632533922	
pals-14	5.4799171274	
F20D6.2	5.4230168305	
clec-228	5.3955474278	
F59F5.4	5.3508101520	
cest-35.2	5.3465219765	
T20G5.12	5.3371693309	
clec-20	5.3225273669	
Y38C1AB.7	5.3211420212	
C28D4.10	5.3125654231	+
arrd-11	5.2889661050	
dmd-10	5.2745215481	
W09C2.8	5.2219497769	
F59A6.10	5.1905195233	
C17G10.6	5.1494415475	
F32D8.8	5.1383349722	+
zip-8	5.1122397223	
T26C12.2	5.0983067488	
lys-3	5.0976135337	
C40H1.9	5.0657427325	
lys-10	5.0499377824	+
mtl-1	5.0172234098	
F20C5.7	5.0112879685	
pals-37	4.9883932857	
M02E1.4	4.9845582009	
W04G5.10	4.9232702547	

pals-26	4.8730112625	
dos-3	4.8728987271	
fbxa-164	4.8359773779	
Y9C9A.16	4.8267664688	
F15H10.10	4.8149568962	
col-151	4.7704875442	
his-34	4.7692471694	+
pqn-97	4.7588638195	
pals-39	4.7582260140	
arrd-28	4.7458374862	
his-29	4.7220416750	+
Y65B4BR.1	4.7137366522	
F26F2.1	4.7113889545	
C12D5.5	4.6997609112	
C29A12.6	4.6853620818	
C17C3.3	4.6796286640	
pals-33	4.6739556378	
Y34F4.2	4.6710642527	
col-86	4.6366883918	
col-116	4.6329221028	
Y47H9A.1	4.6227278930	
cut-5	4.6192877895	
grl-3	4.6192204635	+
clec-61	4.6161883157	
F45D11.16	4.6054984529	
F23B2.10	4.5536430772	
oac-55	4.5419444790	
pals-27	4.5041428815	
col-33	4.5032127744	
tba-8	4.4967102288	+
F07G11.4	4.4904482255	
Y116F11B.9	4.4881245622	
R07C12.1	4.4871107972	
F16B4.7	4.4848737567	
cut-6	4.4555476377	+
W01A11.7	4.4520524555	
F49H6.5	4.4332558117	
sod-3	4.4232749900	+
F53H2.1	4.4224758506	
ZC196.2	4.4143484475	
T22F3.11	4.4061918144	
W07A12.4	4.4016952748	
lips-8	4.3997577649	
C06E7.88	4.3719511629	
M60.7	4.3718175611	+
clec-11	4.3705989812	
F48F7.3	4.3521309721	
col-171	4.3396582497	
C36B7.4	4.3349689403	
mltn-6	4.3235464638	
srh-146	4.2954778334	
fbxa-21	4.2751897153	
F45D11.14	4.2726520541	
F45D11.15	4.2390029616	
T08E11.1	4.2158877601	
Y43F8B.23	4.1638696850	

F10C2.7	4.1436286926	
col-87	4.1346914817	
cyp-35B1	4.1300320937	
his-28	4.1049486208	+
C13A2.1	4.0977473792	+
rgba-1	4.0908354092	
pals-30	4.0908354092	
his-17	4.0511881737	+
math-42	4.0421045719	
W03G1.5	4.0367017594	
Y110A2AL.4	4.0299145976	
M05D6.8	4.0295926585	+
T12D8.5	4.0267636132	
his-50	4.0055018228	+
his-1	4.0017201240	+
C48B4.13	3.9823302693	+
C50F7.5	3.9759365454	
pals-38	3.9736115567	
clec-234	3.9648606914	
ZK218.3	3.9637208552	
F38B7.11	3.9586994888	
F42C5.3	3.9470259117	+
clec-55	3.9441204263	
C05G5.7	3.9271932956	
lact-6	3.9212947298	
W03F9.4	3.9092448149	+
T14A8.2	3.9001503126	+
ref-2	3.8986891608	
his-27	3.8916853069	+
fbxa-138	3.8893222602	
arrd-16	3.8788703307	
C31H5.1	3.8734965673	
his-32	3.8570123101	+
F11A5.17	3.8475316490	
M03A1.3	3.8280054019	
Y73B6BL.37	3.8273802533	
F10B5.3	3.8258596143	
F13E9.8	3.8132297945	
his-49	3.8112115493	+
F14F3.4	3.8010254424	
Y47D3B.3	3.7815035676	
ZC443.3	3.7753188764	+
clec-9	3.7150948301	
srh-2	3.6925430627	+
hpx-2	3.6639114091	
pqn-16	3.6600915462	
otpl-6	3.6600915462	
lipl-4	3.6288269273	
Y43F8B.15	3.6210494792	
ftn-1	3.6203083874	
C25F9.2	3.6070644759	
W04G5.9	3.6056407779	
lmd-4	3.6016484947	
ZC443.2	3.5984520965	+
F44A2.7	3.5928874034	
T14B1.1	3.5918964742	

icl-1	3.5862175592	+
znf-782	3.5796938488	+
asp-16	3.5744420451	
F01D4.8	3.5672428550	
K06C4.1	3.5520137486	
dlhd-1	3.5473850370	
C17D12.3	3.5297149989	
aqp-6	3.5259141264	
cest-7	3.5232169868	
ifas-2	3.5206996687	
T20D4.12	3.4947787583	
C35A5.11	3.4938534200	+
F12A10.9	3.4721486190	
cyp-13A3	3.4715656217	+
gst-3	3.4710925082	
tsp-17	3.4615580698	+
ins-11	3.4568076262	+
cutl-23	3.4525052190	+
Y47H10A.3	3.4459699340	
cng-3	3.4424804882	
clec-34	3.4351789867	
T10C6.15	3.4340218826	
F20B6.4	3.4243030263	
srw-85	3.4168025452	
pals-2	3.4147524019	
W03D2.9	3.4137286767	
T06E4.7	3.4013654660	+
W09G12.8	3.3917732950	
his-11	3.3814475429	+
srx-128	3.3812486864	
clec-17	3.3799787587	
Y18H1A.14	3.3706181416	
T05E11.8	3.3553642176	
his-15	3.3534005218	+
irld-34	3.3516778417	+
cyp-13A8	3.3508541602	
oac-40	3.3496298148	
ugt-4	3.3461231270	
R04B5.6	3.3414207191	
dod-3	3.3405718641	
W08E12.2	3.3258915888	
F14H8.2	3.3249194617	
fbxa-66	3.3190237151	
dach-1	3.3093575839	
fbxa-182	3.3041505099	
cpr-1	3.2979664717	
F26G1.3	3.2976207002	
C17F4.12	3.2864720735	
clec-15	3.2858853645	
sre-6	3.2841968884	
fbxa-136	3.2790594357	
clec-13	3.2764535554	
K03D3.2	3.2732690877	+
gcy-8	3.2730235799	
T26H5.8	3.2724865475	
nhr-221	3.2716080645	

his-44	3.2663783946	+
Y39G8B.5	3.2521734441	
txt-3	3.2443211168	
F10A3.1	3.2431358480	
nhr-30	3.2369918948	
F09F7.6	3.2227360987	
ZK185.4	3.2214738563	
cyp-23A1	3.2209983602	
F09C6.3	3.2168504894	
F32G8.3	3.2080845757	
clec-78	3.2047677818	+
pqn-98	3.1991463738	
Y39A1A.9	3.1975733785	
rab-11.2	3.1964239045	
tsp-6	3.1925701866	
his-22	3.1856971121	+
F32D8.12	3.1711368734	
hil-1	3.1700743842	
mls-2	3.1688939057	
C08E3.13	3.1662561693	
F40F9.10	3.1624040220	
R07C12.3	3.1549342933	
nspg-5	3.1499819187	
T23F6.5	3.1480907571	+
irld-2	3.1440956465	
atf-8	3.1432164351	
his-45	3.1390635327	+
gpa-13	3.1290163187	
T07G12.4	3.1252684870	
nhr-159	3.1189667872	
cyp-35B2	3.1189084631	
ugt-53	3.1170411926	
his-6	3.1082607580	+
cyp-13A1	3.1060991661	
spp-2	3.1058557118	
sodh-1	3.0993971096	+
his-55	3.0978911893	+
cpr-4	3.0918506931	
hen-1	3.0916851505	+
clec-60	3.0877655546	
ZK856.14	3.0868606562	
nhr-21	3.0761231117	+
R06A10.1	3.0745285413	
ugt-59	3.0708864806	
cut-3	3.0673905901	+
F09E10.6	3.0671693813	
B0310.3	3.0624623721	
npax-3	3.0614531667	
eol-1	3.0605078516	
fkb-7	3.0599709652	+
zig-3	3.0542214268	+
T07H8.11	3.0533268615	
sel-7	3.0506028297	+
dyf-19	3.0494954598	+
his-59	3.0491592797	+
W04G5.8	3.0411459228	

F32D8.2	3.0390618039	
F07C4.6	3.0384435763	
nhr-137	3.0370369330	
kreg-1	3.0361656453	
DH11.2	3.0281324932	
T19C9.8	3.0232888255	
F33H12.7	3.0196056308	
R07A4.3	3.0147033126	
C09G5.13	3.0142439885	
mltn-1	3.0086008027	+
his-2	3.0082117663	+
F14D7.10	3.0082117663	
F43C11.8	3.0068342998	
F48C1.11	3.0023346554	
his-14	2.9980402507	+
his-4	2.9975336729	+
his-62	2.9881354277	+
his-10	2.9862991687	+
K03B4.8	2.9861143438	
Y39B6A.1	2.9753941132	
Y41D4A.1	2.9625021305	
E02H4.4	2.9585866801	
F37A4.6	2.9577244467	+
K02C4.2	2.9543791244	+
his-18	2.9469998784	+
R05A10.7	2.9452990077	
nhr-147	2.9345339125	+
C09B8.4	2.9345339125	
F32D8.11	2.9326997393	
ZC395.5	2.9272408774	
ZC204.12	2.9204510997	+
oac-56	2.9197543381	
E04D5.4	2.9146960358	
math-34	2.9112633441	
F37A8.5	2.9108921344	+
ZK105.13	2.9096341922	
EEED8.2	2.8977457047	
Y51B9A.9	2.8941783347	
mpst-2	2.8881260707	
skpo-2	2.8817333804	+
T20D4.11	2.8806944444	
his-63	2.8683524968	+
sqst-3	2.8633890053	
cky-1	2.8628647778	
F21C10.10	2.8617919333	
C04A11.5	2.8613249550	+
ZC239.14	2.8598637019	
skr-5	2.8509125356	+
bnc-1	2.8492268884	
his-12	2.8485498440	+
T22B7.3	2.8469392437	+
peb-1	2.8419828793	+
ZK1240.1	2.8392902595	
F07B7.8	2.8392902595	
cyp-37B1	2.8170845559	
clec-42	2.8138395623	

cutl-9	2.8113969501	+
his-9	2.8093566389	+
ceh-16	2.8086815753	+
ZK836.3	2.8057401494	
C23H5.15	2.7948919135	
his-52	2.7927052626	+
his-25	2.7922138151	+
his-13	2.7912199217	+
Y22D7AL.16	2.7848857641	
M01B2.13	2.7794366875	
ddo-2	2.7781551510	
T12E12.6	2.7757245755	
oac-37	2.7741188705	
C12D5.3	2.7692211246	
his-5	2.7645756803	+
T01B10.5	2.7576677552	
F47B10.8	2.7555905215	+
F17B5.1	2.7370963108	
T10G3.8	2.7367954728	
F47B8.4	2.7304856149	
anoh-1	2.7238700391	
F32D1.11	2.7211200839	
prdm-14	2.7120621736	+
C39D10.5	2.7120621736	
fbxa-52	2.7120621736	
ZK993.2	2.7120621736	
Y116A8C.43	2.7086642770	
fipr-24	2.6964778529	
bro-1	2.6940445742	
srd-53	2.6905152349	
his-16	2.6882412501	+
fbxa-24	2.6845561696	
T05C1.3	2.6824903426	+
his-54	2.6763299861	+
C38D9.2	2.6583927339	+
his-43	2.6545992817	+
C18H9.1	2.6533237454	
B0507.6	2.6475117128	
scl-2	2.6399235041	
his-60	2.6378475408	+
clec-12	2.6349373946	
his-46	2.6200542354	+
his-56	2.6196170880	+
Y69H2.10	2.6168045307	
F47B10.3	2.6145108119	
fbxa-166	2.6136859578	
F44A6.5	2.6125573149	+
B0205.4	2.6107546346	
btb-16	2.6084386878	
T05A8.8	2.6063258865	
C26B9.7	2.6004273087	+
C14A11.2	2.5991460617	
nnt-1	2.5942214772	+
nhr-260	2.5938218784	
his-20	2.5932743963	+
far-7	2.5859547767	

F23H12.5	2.5856562868	
C03B1.5	2.5772435493	
ins-35	2.5717526244	+
acr-14	2.5706628366	
rol-8	2.5706006353	
T07G12.5	2.5688058958	
F26D11.2	2.5681964546	
dhc-3	2.5677643754	
ast-1	2.5659551712	
trp-4	2.5628759415	
hacd-1	2.5614801588	
T19C3.3	2.5581183238	
F42G2.5	2.5546369309	
F49C12.10	2.5545664591	
cla-1	2.5542519287	
sre-4	2.5532538881	
Y46G5A.38	2.5514127285	
C43H6.6	2.5509686513	
his-66	2.5490982904	+
dpy-1	2.5477415192	+
slc-36.5	2.5458460904	+
gcy-36	2.5456903616	
W09C2.7	2.5433979925	
lmd-5	2.5388527471	
C35A11.2	2.5385364285	
Y34F4.6	2.5345958294	
comt-1	2.5312261326	
cyp-14A1	2.5286136949	
his-58	2.5250556015	+
H40L08.3	2.5240900265	+
cbn-1	2.5197329870	+
ugt-57	2.5189085323	
mfb-1	2.5184795433	
pag-3	2.5144517270	
oac-14	2.5125216729	
clec-145	2.5118303786	
C44F1.1	2.5111785879	
fbxa-163	2.5042706137	
F15B9.6	2.4968892948	
K03A11.4	2.4957693253	
his-65	2.4933350807	+
math-37	2.4911231125	
T06G6.6	2.4875889837	+
K02A2.5	2.4860381977	+
F57B9.3	2.4847706948	
gpdh-1	2.4841217674	+
ZK1225.1	2.4685264193	
his-26	2.4682001420	+
B0507.8	2.4653501072	
his-48	2.4642762973	+
kgb-2	2.4618880717	
C28C12.4	2.4600504012	
nhr-208	2.4590696963	+
C15F1.2	2.4576839541	
Y110A2AL.3	2.4555148541	
his-47	2.4509077293	+

F47F2.3	2.4479533162	
pals-31	2.4465860870	+
his-57	2.4442672866	+
Y71H2AM.25	2.4430513416	
F26G1.9	2.4395047112	+
lim-4	2.4365639390	
col-46	2.4187480409	
Y6G8.2	2.4147108052	
F39F10.4	2.4064563309	+
F53A9.7	2.4039768414	
F32D8.3	2.4035891819	+
Y6E2A.5	2.4031428624	
F15E6.3	2.4022018168	
dct-1	2.4016876401	
K01A2.4	2.3964880149	
C54C8.4	2.3958306964	
Y49G5A.1	2.3946795222	
C54E4.12	2.3946795222	
T20D4.10	2.3916306277	
H41C03.1	2.3914262166	+
lys-4	2.3892643115	+
col-128	2.3884742206	
pef-1	2.3855957336	
C09G1.2	2.3828790544	
C06B3.6	2.3732685731	+
slc-36.3	2.3716439740	+
ZC13.10	2.3708834672	
cutl-14	2.3675649118	+
F20A1.6	2.3668343822	+
T04C12.8	2.3635632324	
C02G6.3	2.3619589457	
K09C4.4	2.3614111123	
mam-5	2.3609539691	
unk-1	2.3583999634	
gcy-3	2.3553093991	
lag-2	2.3551011637	+
K09F6.11	2.3551011637	
tax-2	2.3505221167	
F26F12.3	2.3453436896	
nas-23	2.3414832324	
ugt-52	2.3403840238	
C17F4.2	2.3384737091	+
mltn-3	2.3335200894	
mlt-11	2.3327962272	
cdh-7	2.3306483894	
Y46G5A.36	2.3299751708	
ZC443.4	2.3294718494	
C30B5.6	2.3288449053	
K09F6.10	2.3281364692	
K02H11.4	2.3273765150	
npax-1	2.3258582311	+
F26G1.10	2.3255300982	
C34F6.1	2.3252494152	
ZK54.3	2.3243932679	
F33H2.8	2.3241470380	
C05C8.8	2.3209931414	

col-176	2.3209720923	
odr-1	2.3201754551	
K10C9.1	2.3201754551	
fut-2	2.3170652982	
best-5	2.3156212335	
srg-7	2.3155882891	
hil-7	2.3136625806	+
acdh-2	2.3086482336	
grl-22	2.3078294969	
F57G12.2	2.3039105184	
R11G1.7	2.2999931889	+
acdh-1	2.2996829474	+
F35F10.1	2.2987133546	
Y110A2AL.9	2.2978235762	
aat-8	2.2925483972	
col-89	2.2894731639	
nlp-2	2.2887596935	
pals-1	2.2887250351	
C15C8.8	2.2887111737	+
lgc-1	2.2887111737	+
B0462.5	2.2872964350	
F38E11.6	2.2856362742	+
zmp-2	2.2849107617	+
cdh-10	2.2847767177	
del-8	2.2841644919	
T01D3.3	2.2840786037	
T17H7.1	2.2826254610	
acs-21	2.2819626869	
dpy-9	2.2808587434	
C39B5.14	2.2796756784	
tyr-1	2.2796367427	+
ptr-10	2.2786479532	+
bus-1	2.2780565195	
aptf-1	2.2773193488	
F47H4.2	2.2769500652	
twk-7	2.2766811583	
K09H11.6	2.2755733131	
ubc-8	2.2750625660	
F52C9.5	2.2742605661	
T04H1.12	2.2729113327	
ddn-1	2.2721499576	
C06G3.3	2.2706492851	
F35D2.6	2.2685753062	
C07A4.2	2.2631435585	
grl-10	2.2626164151	+
fbn-1	2.2621135832	
srd-49	2.2612732331	
T10G3.1	2.2587682036	
EGAP4.1	2.2568276271	
Y46E12BR.1	2.2560217382	
C07F11.2	2.2557574139	
T28F4.5	2.2556406284	+
rml-3	2.2528300133	
R04E5.7	2.2515786055	
Y2H9A.4	2.2503006160	
myrf-1	2.2500578947	+

scl-5	2.2472657916	
Y37A1B.7	2.2428023247	+
nhr-113	2.2427917870	
K08F9.1	2.2407204338	
lipl-2	2.2397615242	
B0554.7	2.2390951276	
W09G12.7	2.2361384449	
his-7	2.2358171834	+
maph-9	2.2349582985	+
clec-72	2.2336828506	
math-28	2.2333291197	
B0294.1	2.2314835948	
lpr-1	2.2312624094	+
T21B4.15	2.2308737788	
fkh-7	2.2306770330	
T22F7.3	2.2295445806	
C05E11.7	2.2293894901	
F48G7.10	2.2267503808	+
T02G5.3	2.2252412122	+
T04C12.7	2.2235057618	
F26A1.8	2.2234232841	
R03H10.2	2.2228749765	
droe-4	2.2169954775	
T27F6.8	2.2163551154	+
C54D10.9	2.2161855079	
Y71G12B.32	2.2147025415	
ztf-14	2.2135530940	
F54B11.10	2.2121314874	
ZC84.6	2.2111610274	
fbxa-30	2.2103892206	
K06A9.2	2.2078008819	
F20C5.5	2.2020196727	+
F09C6.11	2.1947352358	
clec-169	2.1942125835	
Y4C6B.4	2.1940024791	
clec-47	2.1935839562	
C06C3.12	2.1899966112	
daao-1	2.1881077802	
nhr-19	2.1852061255	
C30F2.4	2.1841963847	
dod-20	2.1835545695	
his-64	2.1833983515	+
nhr-263	2.1796474090	+
T20B3.1	2.1795846271	
C17G10.7	2.1792089481	+
fbxl-1	2.1783229968	+
nhr-157	2.1762281293	
lgmn-1	2.1761360774	
T08G5.15	2.1757627398	
C48D5.3	2.1736987897	
C17F4.3	2.1695889510	
Y105E8A.13	2.1673801012	+
C08D8.1	2.1662611786	
lys-7	2.1662331087	+
aakg-4	2.1651705419	
F57C9.6	2.1625795876	+

nas-4	2.1619733944	+
Y73B6BL.44	2.1614313966	
tsp-1	2.1603451033	
glb-21	2.1582754915	
F13G3.3	2.1535106452	
acer-1	2.1520368438	+
fat-5	2.1485948155	
spp-18	2.1474003899	
T12A7.6	2.1461467758	
R07E3.6	2.1460986072	
nhr-150	2.1456102571	
F42C5.4	2.1445171734	
cup-16	2.1431391617	
let-4	2.1428546910	
C08F11.3	2.1414852618	
R05H5.7	2.1409566472	
igdb-3	2.1401893430	+
C25E10.16	2.1399101245	
tsp-2	2.1380267344	
F47B8.2	2.1369889194	
F21H7.12	2.1360138933	+
ztf-30	2.1352461083	
F28G4.2	2.1345898519	
clec-82	2.1343130569	
Y23H5B.3	2.1333341951	
arl-3	2.1293446765	
dpy-7	2.1280993019	
wrt-2	2.1263242492	
T14B4.19	2.1214765465	
R13H4.7	2.1174564280	
igcm-1	2.1167367759	
K02E10.5	2.1158035388	
his-21	2.1138129048	+
F44G3.10	2.1124946094	
hsp-12.3	2.1114617620	
clec-194	2.1097512979	
W08E12.3	2.1079336281	
C27H5.6	2.1077641857	+
ZK757.1	2.1052528258	
dhs-16	2.1045015150	
sru-40	2.1009598220	
Y65B4BL.1	2.1002766203	+
C45G7.4	2.0979512952	+
K02E10.4	2.0957493217	
dpy-8	2.0949820493	
K02F6.8	2.0941927383	
frpr-10	2.0941927383	
acc-1	2.0934299475	+
oac-29	2.0910069059	
F35D2.1	2.0906578903	
Y44A6C.1	2.0898692106	
ZK971.1	2.0877245084	
W03D8.8	2.0871878992	
Y43C5A.3	2.0869477994	
C09F9.2	2.0859643287	
nlp-52	2.0818294871	

dpy-10	2.0807023821	+
ceh-2	2.0796515028	+
nhr-227	2.0796515028	
F59G1.4	2.0784703624	+
atf-2	2.0778348155	
otpl-5	2.0767327158	
ttr-23	2.0757595556	
fbxa-158	2.0731835404	
F20C5.6	2.0702871661	+
fkh-8	2.0699887203	
faah-6	2.0688921572	
Y54G2A.76	2.0683825217	
twk-11	2.0679153445	
F01G10.6	2.0653317240	
ZC449.2	2.0650589740	+
ztf-2	2.0617471934	
F25E5.8	2.0593689213	+
wht-1	2.0591755212	
oac-24	2.0579598205	
C06E4.8	2.0569468283	+
igcm-2	2.0536678721	+
mltn-8	2.0532439356	
E04F6.9	2.0522750021	
Y41G9A.5	2.0521487722	
marc-5	2.0518473864	
math-15	2.0468956945	
cest-29	2.0468956945	
tag-272	2.0448050941	
bus-12	2.0447473102	
talp-3	2.0417601545	
hil-3	2.0413895383	+
C15C8.5	2.0411656681	
fozi-1	2.0405655276	+
dpf-6	2.0378321278	
T13C5.3	2.0369924593	+
H03E18.1	2.0361361166	
trpl-3	2.0339552952	
K10D3.4	2.0333957554	
col-172	2.0307793706	
osr-1	2.0303629223	
lron-1	2.0301874687	
clec-75	2.0282557538	
K10H10.5	2.0280001182	
C14A4.9	2.0265512716	+
R03H10.7	2.0263001533	+
clec-180	2.0262390626	
nhr-68	2.0254352939	
C15A7.2	2.0252427859	
C29F9.2	2.0248688737	
M01H9.3	2.0221375622	+
ZK593.2	2.0218210480	
F57F5.3	2.0211232383	+
B0348.2	2.0193690876	
noah-2	2.0179281231	
F13H6.5	2.0158831310	
cutl-21	2.0135019565	

T26C5.2	2.0113781791	
col-111	2.0071525906	
spi-1	2.0071351283	+
lpr-5	2.0070436921	
F45D3.4	2.0067508087	+
dgk-3	2.0065024720	
Y64H9A.2	2.0045578060	+
dex-1	2.0027527073	
R11D1.2	2.0011049682	
sup-26	2.0007826429	+
pzf-1	-11.1774448148	+
F43G6.7	-10.2633281267	+
vet-2	-10.1308033891	+
M151.3	-9.6544046392	+
T25E12.6	-9.4298331635	+
nspe-6	-9.3768002659	
F31F6.1	-9.3029138949	+
T24E12.1	-9.2645014786	+
Y67D8B.3	-9.0997265377	
skr-15	-8.8631568291	+
btb-7	-8.8631568291	+
fbxc-42	-8.8631568291	+
F17A2.13	-8.8372179490	+
Y71A12B.23	-8.8108041499	
ceh-40	-8.8108041499	+
C45H4.14	-8.6709542721	+
B0281.5	-8.6709542721	+
enri-2	-8.5484121234	+
skr-13	-8.5484121234	+
F02D10.6	-8.5160782207	+
gadr-2	-8.4830029993	+
C47F8.1	-8.4491516677	+
C46E10.8	-8.4491516677	+
F26G5.1	-6.5215988549	+
F12E12.1	-6.1538236661	+
vit-4	-5.9935456841	+
vit-3	-5.8797465248	+
F19H6.4	-5.8275202988	+
K04C1.5	-5.7843894820	+
W06D11.3	-5.6073442481	+
vet-1	-5.5227437483	
skr-7	-5.4362779396	+
skr-14	-5.4244747282	+
C17E7.4	-5.4231019319	+
spp-9	-5.4132165616	
T02G6.5	-5.3417345260	+
sea-1	-5.3066529512	+
nhr-2	-5.2875593676	+
tbx-33	-5.2632531735	+
F23D12.2	-5.2485969669	+
ceh-49	-5.2214397080	+
ztf-25	-5.1681058736	+
meg-1	-5.1630464169	+
F22E5.17	-5.1034300489	+
ZK177.1	-5.0583494280	+
R04D3.3	-5.0447251073	+

F53F8.3	-5.0413664034	+
meg-4	-4.9991678730	+
R04D3.4	-4.9965275296	+
fkh-3	-4.9830693602	+
sri-40	-4.9282444734	+
Y47D7A.7	-4.8919963894	
fbxa-170	-4.8792988203	+
Y45F10C.2	-4.8669746296	
cdc-25.3	-4.8032298663	+
rgs-11	-4.7650235855	+
C01G6.3	-4.7486386227	+
Y53G8AM.4	-4.7460875262	
C31G12.1	-4.7452483472	+
C49F5.3	-4.7136882337	+
F54F7.6	-4.6888702331	+
fbxc-32	-4.6886013864	+
dod-21	-4.6816682629	
F22F4.5	-4.6694197939	
vit-1	-4.6463114377	+
C04B4.2	-4.6419930617	+
ent-5	-4.6341622665	+
Y75D11A.3	-4.6073368299	+
ccch-5	-4.5713231728	
C32H11.9	-4.5496800065	
Y57G11B.5	-4.5263757701	
vit-5	-4.5193713066	+
dod-23	-4.4731902387	+
F02E8.4	-4.4557792139	+
C17E7.12	-4.4347147307	+
F14H3.6	-4.4275385940	+
rgs-9	-4.4213025806	+
ssp-19	-4.4179515416	+
F14H3.3	-4.3961166559	+
R09F10.8	-4.3796469563	+
F08F3.6	-4.3354014800	+
btb-6	-4.3077954621	+
F56D6.12	-4.2837592825	
meg-2	-4.2690786513	+
ugt-63	-4.2321801170	+
col-143	-4.2200246044	
nos-2	-4.2172268064	+
lsy-27	-4.2033589019	+
ttr-42	-4.1905535208	+
Y62H9A.3	-4.1861682490	
F40G12.11	-4.1833517001	+
T12G3.6	-4.1691701320	+
C08F11.12	-4.1577868350	+
F31F6.2	-4.1561648155	+
F31B9.3	-4.1391411618	+
Y41C4A.32	-4.1353933186	
F31F6.3	-4.1334492697	+
tbx-36	-4.1333316875	+
Y47D7A.6	-4.1262639282	
T24E12.11	-4.1262639282	+
Y62H9A.5	-4.1206067583	+
tbx-9	-4.1142879654	+

K03A11.1	-4.1049481388	
ule-5	-4.0747783353	+
F14H3.4	-4.0655692738	+
W02D9.6	-4.0514840802	
C50E3.12	-4.0507097543	+
F15A4.10	-4.0145799353	+
R05G9.3	-4.0118468562	+
C50E3.11	-4.0111444176	+
M151.7	-4.0001688007	+
nspb-4	-3.9979798300	+
F14H3.5	-3.9815615586	+
Y17G7B.19	-3.9758648924	
E02H4.6	-3.9691717190	+
rgs-8.2	-3.9681515514	
rgs-8.1	-3.9502430491	+
Y25C1A.14	-3.9477598034	
T04D3.1	-3.9443185563	+
meg-3	-3.9432423472	+
puf-5	-3.9418253568	+
T06D4.1	-3.9406935892	+
oac-20	-3.9333189408	
F14D7.2	-3.9078952898	+
C27C12.3	-3.9061755736	+
ZK829.9	-3.9057039619	+
Y105C5B.20	-3.8935191344	+
msrp-5	-3.8605208958	+
fbxc-50	-3.8538622633	+
T01G5.7	-3.8333096232	+
C46C2.5	-3.7987387064	+
R09A8.2	-3.7837127712	+
nas-27	-3.7725123255	
R09A8.1	-3.7695233186	+
try-1	-3.7674181319	+
C50E3.13	-3.7640536911	+
F19B10.5	-3.7474439558	+
F02H6.2	-3.7442256027	+
F39F10.3	-3.7311518831	+
clec-91	-3.7125635486	+
hrg-4	-3.7078930972	+
oma-2	-3.6812680964	+
B0513.4	-3.6811584477	+
F43G6.10	-3.6799822369	+
clec-87	-3.6798426507	+
fkh-4	-3.6758358394	+
perm-2	-3.6661249399	+
F49F1.7	-3.6365555519	
egg-1	-3.6310050837	+
pos-1	-3.6248474318	+
ule-3	-3.6160973921	+
ule-1	-3.6145763851	+
Y49F6C.8	-3.5968826578	+
R04D3.2	-3.5906460588	+
Y51F10.2	-3.5711492836	+
Y66A7A.9	-3.5532936906	
C17E7.9	-3.5523342654	+
K08H2.3	-3.5374546715	+

F02H6.3	-3.5340470364	+
B0416.4	-3.5128181174	+
K04G2.10	-3.5061457201	+
Y54F10AM.11	-3.5021924974	+
vit-2	-3.4912748708	+
cyp-13B2	-3.4878845665	+
F42A10.7	-3.4873687493	
ZC373.2	-3.4872090154	
msp-63	-3.4868185111	+
W02D9.7	-3.4786288080	
D1054.10	-3.4758012779	+
drd-50	-3.4631137428	
19H5.7	-3.4540332349	
cpg-1	-3.4416082457	+
T01C3.3	-3.4313282764	+
K10C2.8	-3.4302413145	+
msrp-6	-3.4277548274	+
msp-49	-3.4259157466	+
C35E7.5	-3.4227141895	+
era-1	-3.4152081936	+
rme-2	-3.4036931691	+
flh-3	-3.3991329705	+
gln-5	-3.3979437123	+
col-8	-3.3977458210	+
cpg-2	-3.3925823244	+
perm-4	-3.3914309966	+
gfat-2	-3.3885183552	+
msp-50	-3.3871602105	+
Y53H1C.3	-3.3859107046	
F54D10.7	-3.3808592826	+
cpg-3	-3.3797979056	+
T15H9.5	-3.3744162157	
ZC412.8	-3.3733648480	
W05F2.3	-3.3730085882	+
scl-17	-3.3703196954	
nspe-1	-3.3559520196	
col-178	-3.3519573293	+
C05C10.5	-3.3506590140	+
H12D21.3	-3.3487534982	
col-20	-3.3476986063	+
K09E3.7	-3.3194920403	+
col-137	-3.3096226744	+
ceh-83	-3.3014332912	+
C01G8.1	-3.2954135406	+
puf-3	-3.2953127976	+
col-119	-3.2897527378	+
ZK813.1	-3.2830469633	
ZK813.3	-3.2771905405	
ZC190.7	-3.2730227804	
clec-97	-3.2729596845	
Y37D8A.19	-3.2592806148	+
Y62H9A.4	-3.2563647815	+
ZC308.4	-3.2527660911	+
mex-1	-3.2466605892	+
ssp-33	-3.2464224871	+
vit-6	-3.2367408169	+

nspg-7.2	-3.2281980268	
BE10.5	-3.2189901082	
chs-1	-3.2134174953	+
C24D10.5	-3.2081075866	+
T20B6.1	-3.1914218039	
F08B4.8	-3.1910169167	
msp-38	-3.1887677540	+
T21C9.13	-3.1863760093	+
col-181	-3.1828401867	+
egg-3	-3.1821654172	+
C45B2.1	-3.1749035402	+
gyg-2	-3.1721911633	+
W06D11.1	-3.1656571697	+
nspc-2	-3.1629117705	
col-179	-3.1619609329	
egg-5	-3.1590712539	+
F08H9.2	-3.1537863820	+
ssp-10	-3.1496284082	+
memi-3	-3.1483004267	+
col-101	-3.1453615303	+
K08C9.1	-3.1451787101	+
F36D1.4	-3.1411810336	
EEED8.3	-3.1231406369	+
Y4C6A.3	-3.1213828989	+
msp-65	-3.1207249845	+
msp-58	-3.1115679607	+
C10G8.4	-3.1077775123	+
msp-64	-3.1029743840	+
col-122	-3.0934352907	
cyb-2.2	-3.0918937257	+
col-140	-3.0882223557	
F58A6.9	-3.0833479223	
col-80	-3.0802756965	+
puf-11	-3.0776611955	+
C30G12.2	-3.0760222242	
col-96	-3.0712689214	
C04H5.7	-3.0675269940	+
T10B5.8	-3.0648261667	
egg-2	-3.0563644857	+
F53F4.18	-3.0545144953	
lido-12	-3.0543205355	+
ssq-1	-3.0521096423	+
Y39E4B.5	-3.0487950911	+
mesp-1	-3.0478836942	+
col-142	-3.0432025404	
col-124	-3.0385009029	+
mrac-3	-3.0245255086	+
T10C6.7	-3.0217708187	+
F55B11.3	-3.0178868614	+
msp-76	-3.0166694515	+
T09B4.3	-3.0113874932	
ht-3	-3.0042100846	+
T22B7.7	-2.9994538638	
mex-6	-2.9990230006	+
nspc-5	-2.9941897842	
F10D11.3	-2.9926401377	+

clec-88	-2.9911382635	+
C29F7.10	-2.9901652087	+
msp-152	-2.9868148646	+
F32D1.7	-2.9824727734	+
nep-7	-2.9816920808	
nsph-4.1	-2.9694438069	
clec-175	-2.9626497281	+
msrp-4	-2.9583976169	
F48E3.4	-2.9573154700	+
C06C6.7	-2.9459181580	+
T27A8.2	-2.9455763417	+
F22E5.1	-2.9423691040	
gln-6	-2.9383360692	+
nspd-2	-2.9382598092	+
msp-78	-2.9377192272	+
col-19	-2.9355248066	
ZK813.2	-2.9295753958	
msp-113	-2.9291854688	+
R102.8	-2.9246071935	+
ZK39.10	-2.9239203560	
col-184	-2.9223342175	
ZK813.7	-2.9192650039	
T16G1.13	-2.9180804311	
fbxb-67	-2.9170752108	+
msd-4	-2.9022231519	+
puf-7	-2.8994794145	+
Y71F9B.15	-2.8982071873	
H08M01.1	-2.8961247141	+
msp-59	-2.8959363772	+
C10A4.4	-2.8933727878	+
ssp-34	-2.8868768232	
nspd-10	-2.8848366822	
nspc-6	-2.8846107912	
col-93	-2.8761579404	
puf-6	-2.8704686705	+
Y38C1AA.7	-2.8685230114	
kca-1	-2.8676140674	+
spn-4	-2.8668186649	+
msp-33	-2.8667490803	+
nspd-4	-2.8643831134	
msp-45	-2.8604437841	+
skpo-1	-2.8547272490	+
W10G11.3	-2.8491714695	
msp-3	-2.8472284256	+
nspe-2	-2.8471751271	+
T11F8.1	-2.8448125477	+
gna-2	-2.8430123676	+
msp-77	-2.8426011828	+
C39D10.7	-2.8400677470	+
col-106	-2.8388910487	
F18A1.7	-2.8363943546	+
msp-36	-2.8250078044	+
ZK402.5	-2.8227956663	+
ssp-9	-2.8199359948	+
msp-10	-2.8185400383	+
ssq-2	-2.8170039212	

K07A1.6	-2.8121380989	+
msrp-3	-2.8095233309	+
cbd-1	-2.8089157034	+
Y75B12B.1	-2.8022167887	+
cpar-1	-2.7982284683	+
ssq-4	-2.7961572249	
nspc-1	-2.7960241360	
R03G8.6	-2.7957110258	
F34D10.8	-2.7953005350	+
F36D3.16	-2.7899320535	
col-81	-2.7854878564	
srg-10	-2.7841591848	
F17E9.5	-2.7760771715	+
F55B11.2	-2.7753224019	+
Y48B6A.10	-2.7660440233	+
rmd-1	-2.7654617323	+
ssp-11	-2.7651795879	
Y39F10C.3	-2.7616301842	
Y51B9A.5	-2.7606334017	
abu-2	-2.7606226962	
C11E4.7	-2.7598892415	
col-7	-2.7592776548	
msp-79	-2.7588466196	+
dct-19	-2.7571150879	
nspd-3	-2.7561933627	
msp-51	-2.7548454037	+
T07E3.2	-2.7545485271	
EEED8.15	-2.7489472870	+
H29C22.1	-2.7477803374	+
col-88	-2.7449079583	
B0261.5	-2.7430142101	+
F57C2.4	-2.7401850466	+
F27C1.1	-2.7390188302	+
msp-142	-2.7371569543	+
msd-1	-2.7309270323	+
egg-4	-2.7303846224	+
spsb-2	-2.7290943423	+
nspe-4	-2.7278472039	+
nspd-5	-2.7270058627	+
C10C5.3	-2.7264804489	+
F53B6.4	-2.7251419472	+
mex-5	-2.7249365392	+
Y73B6BL.288	-2.7228533197	+
col-133	-2.7219218825	
sss-1	-2.7187272035	+
C44B7.5	-2.7180654426	+
C33F10.1	-2.7169821460	
R04A9.9	-2.7163643283	
F21H7.5	-2.7112954860	+
Y45F10C.4	-2.7023027981	
irld-18	-2.7012194676	
34I24.1	-2.7001671169	+
nspd-1	-2.6988967082	
tdc-1	-2.6980445221	+
F36H2.6	-2.6939566159	
Y106G6A.4	-2.6938915595	

pqn-90	-2.6936192826	
Y51A2B.6	-2.6933212700	
ssp-37	-2.6924109207	
msd-2	-2.6886839601	
col-129	-2.6860744057	+
T27E7.1	-2.6854609570	+
col-139	-2.6838419470	
nspd-7	-2.6834808913	
msd-3	-2.6822254100	+
ubxn-5	-2.6789048239	
ttr-50	-2.6785468712	+
memi-2	-2.6763809882	+
K03B4.6	-2.6742547707	
T08B2.12	-2.6740302747	
K11D12.13	-2.6730925709	+
W06A7.4	-2.6724518633	+
F26B1.8	-2.6699597614	
Y59E9AR.7	-2.6690017071	+
memi-1	-2.6670946738	+
Y47D3B.12	-2.6661970549	
oma-1	-2.6654302348	+
T24D1.3	-2.6644117420	+
F53H4.2	-2.6626259432	
msp-31	-2.6589888419	+
C48E7.7	-2.6589743152	+
F10C1.3	-2.6579556345	+
szy-4	-2.6575725295	+
trcs-1	-2.6555813436	+
msp-19	-2.6537994870	+
F41E6.1	-2.6522689186	
W06B4.1	-2.6487033550	+
D1086.6	-2.6469886080	
T04G9.7	-2.6467269126	+
K08D8.11	-2.6442074362	
F56D6.19	-2.6442074362	
F59A6.12	-2.6411241189	+
nsph-4.2	-2.6405105356	
C15C6.2	-2.6394643366	+
C34F11.2	-2.6379468366	+
Y59E9AR.1	-2.6343908692	
nspd-6	-2.6239371340	+
tbh-1	-2.6231812592	+
D1086.11	-2.6222303789	+
cyb-2.1	-2.6187883226	+
C17F3.1	-2.6152035506	+
C34D4.3	-2.6145964594	+
perm-1	-2.6144078719	+
clec-218	-2.6093461173	
Y46B2A.4	-2.6075320816	
sysm-1	-2.6028201256	
msp-81	-2.5988194148	+
W03B1.3	-2.5923472831	
C44B9.3	-2.5903255376	+
nsph-3.2	-2.5891792586	+
Y71G12B.33	-2.5842266011	
rpl-42	-2.5795729413	+

T10C6.10	-2.5770444229	+
col-62	-2.5700535547	
Y67D8B.5	-2.5676276764	
F36H12.5	-2.5653292326	+
Y69E1A.1	-2.5583049789	
mpst-6	-2.5549872267	
mpst-5	-2.5513380829	
Y49E10.29	-2.5502760879	
F58B4.7	-2.5437574829	
Y53F4B.24	-2.5419073370	
F55B11.5	-2.5409990613	+
msp-55	-2.5380169031	+
Y18D10A.11	-2.5377829363	+
Y52B11A.8	-2.5372056108	+
F59A1.13	-2.5368556953	
C27D9.1	-2.5359960788	+
nasp-2	-2.5325824779	+
Y39G10AR.16	-2.5324433459	
F49D11.14	-2.5281374440	
W09C3.7	-2.5258754850	
C30G7.4	-2.5239857485	+
D2045.5	-2.5188990959	+
Y39B6A.10	-2.5175498873	+
C45B11.8	-2.5134537696	+
Y38F2AR.10	-2.5129120429	
cey-2	-2.5109868659	+
F36D3.4	-2.5086663668	+
B0025.5	-2.5083518916	
C28D4.7	-2.5042944424	+
B0524.2	-2.5030932029	+
C18G1.9	-2.5025437966	+
F52F12.8	-2.5007035855	+
ZK1307.4	-2.4989318597	
F35H8.4	-2.4971346206	+
nsph-3.1	-2.4965383746	
lido-9	-2.4963790469	+
R09E10.6	-2.4957775375	+
nspb-7	-2.4952373759	
F33D11.1	-2.4947076307	+
ZK938.1	-2.4936772264	
H11L12.1	-2.4882042995	+
parg-2	-2.4864723792	
F13A2.4	-2.4863008962	+
F21F8.5	-2.4788226334	+
mei-2	-2.4776689148	+
F48C1.9	-2.4768309299	
msp-74	-2.4721364068	+
Y51H7C.3	-2.4687729435	+
F41H10.1	-2.4673404471	
abu-9	-2.4629612358	
msp-56	-2.4613310941	+
nep-10	-2.4577997431	+
C53B4.3	-2.4559741127	
msp-53	-2.4516642724	+
ssp-31	-2.4506681536	
col-159	-2.4465809754	

dml-1	-2.4464321392	
E02H9.7	-2.4421661881	+
Y57A10B.7	-2.4387488927	
C05B5.12	-2.4376850622	
C02F5.2	-2.4365761432	+
F55H12.5	-2.4355065520	+
Y106G6H.13	-2.4346290037	
F59H6.15	-2.4334187515	
F54D5.5	-2.4330360940	+
F30A10.12	-2.4287080971	+
C05B5.2	-2.4275321132	+
C14B1.9	-2.4273430126	+
pqn-57	-2.4262832390	
C33F10.11	-2.4231850563	+
fat-7	-2.4200677094	
T05F1.2	-2.4195204436	+
nspb-3	-2.4182463128	
F56D6.14	-2.4170611898	
Y52D5A.2	-2.4165686844	
msrp-2	-2.4150537484	
ZK84.5	-2.4138454017	
K07F5.17	-2.4103840813	+
C04F12.12	-2.4103840813	
nsph-2	-2.4088070030	+
Y6E2A.10	-2.4074674259	
M02H5.8	-2.4069538928	
DY3.8	-2.4060255799	+
ssp-35	-2.4037082291	+
T23B3.5	-2.4023192537	+
T05G5.4	-2.3978950317	+
C10C5.5	-2.3928140896	
nspa-8	-2.3912940447	
F47D12.7	-2.3874218737	+
F52D2.12	-2.3862983179	+
T22A3.12	-2.3822663984	+
F47B3.2	-2.3806430231	+
W03B1.5	-2.3806430231	
F17E9.4	-2.3757669080	+
msrp-1	-2.3685560750	+
mom-2	-2.3666369983	+
F52H3.6	-2.3645142493	+
nlp-25	-2.3640728248	
C32E12.1	-2.3638994477	+
acp-3	-2.3632373089	+
K04G2.4	-2.3610327411	+
F35C5.1	-2.3602852144	+
C50E10.1	-2.3559617100	
fipr-7	-2.3526263657	+
C47A4.5	-2.3515345322	
C08A9.10	-2.3514866192	+
F41G3.5	-2.3480030692	+
C55C3.6	-2.3422511893	
R105.1	-2.3405276283	+
nspa-3	-2.3379091317	
F36A4.5	-2.3377561253	+
T08B6.9	-2.3368156810	

F26F4.2	-2.3360434363	+
F47B8.10	-2.3339941695	+
T04B2.8	-2.3310081556	
Y22D7AR.10	-2.3283787909	+
lec-153	-2.3283406505	
C46E10.1	-2.3255942618	
Y45F10B.3	-2.3247767014	
gipc-2	-2.3232046043	+
M162.7	-2.3191922919	+
T03F6.6	-2.3179940095	
spe-12	-2.3178843747	
msp-57	-2.3175144185	+
fbxa-215	-2.3137854622	+
K01D12.7	-2.3137391041	+
K07A1.5	-2.3131819207	+
ZK849.6	-2.3079468376	
cylc-1	-2.3072763143	+
F36A4.4	-2.3070225434	
dgtr-1	-2.3049976639	+
M02B1.4	-2.3049322479	+
K06A5.2	-2.3036140028	
C09B9.4	-2.3026761563	+
Y53F4B.36	-2.3000979540	
rmd-3	-2.2999008674	+
Y59H11AM.1	-2.2992354842	
C10G11.8	-2.2983034124	+
T23G11.1	-2.2978195201	
T04B2.7	-2.2968442196	
K08A2.2	-2.2967609812	+
smz-1	-2.2946548882	+
T12B5.15	-2.2941295316	
nspa-1	-2.2928497718	
ule-4	-2.2928460692	+
C39H7.1	-2.2926004696	+
nspa-5	-2.2902751724	
nos-1	-2.2893733307	+
Y66D12A.3	-2.2872588299	
27B7.6	-2.2868605900	+
rgs-10	-2.2856207419	+
nduo-3	-2.2837746640	
spe-27	-2.2812393903	
gpd-1	-2.2779952393	+
ZK858.2	-2.2689104582	
ZC53.1	-2.2673148068	+
C50F7.3	-2.2669772999	+
T20B6.2	-2.2660888611	
W03D8.3	-2.2659489802	+
F28A10.7	-2.2652724455	
F36A2.10	-2.2643396864	+
col-141	-2.2634691824	
F36H12.4	-2.2628066204	+
C16A11.7	-2.2619884961	
nspb-1	-2.2585095385	
F20D6.6	-2.2557268052	+
msp-40	-2.2529404085	+
B0261.6	-2.2517353393	+

C47A4.3	-2.2476093390	+
lido-16	-2.2470285308	
Y43F8C.9	-2.2464062509	
F56D6.13	-2.2436198518	
C05C12.5	-2.2432257330	+
nas-20	-2.2431357046	
C46H11.6	-2.2407953424	+
C27D8.2	-2.2397748862	+
K01H12.4	-2.2355760447	+
pie-1	-2.2333559870	+
snb-6	-2.2331681809	
C14C10.1	-2.2321363648	+
dct-9	-2.2274058186	+
efn-3	-2.2246315532	+
R102.10	-2.2226183416	+
Y59H11AM.4	-2.2208951570	
T22D1.5	-2.2198567990	+
E03H12.5	-2.2185956048	+
Y51A2B.5	-2.2183484683	
ZC449.8	-2.2176535390	+
fipr-8	-2.2168972653	
fbxa-147	-2.2158089021	
wdr-5.3	-2.2140239192	+
Y43F8C.5	-2.2124387920	
mex-3	-2.2116045174	+
C14A6.6	-2.2109767312	+
Y41E3.18	-2.2106429051	
acs-15	-2.2081312894	+
C45B2.8	-2.2030138492	
F32H2.7	-2.2017166447	+
F43E2.6	-2.2004714355	+
ddo-3	-2.1981428178	+
K08D12.6	-2.1971900199	+
R08A2.1	-2.1953481006	+
K04H4.5	-2.1952890915	+
F13A7.1	-2.1936843150	+
inx-22	-2.1933791204	+
T16G12.8	-2.1917971138	+
nspb-8	-2.1914816562	
T06A10.107	-2.1894457348	
D2062.1	-2.1885779008	+
C27D8.1	-2.1878123714	+
cyb-1	-2.1872820447	+
R10E9.2	-2.1868020438	+
F46F5.10	-2.1855121272	
Y38H8A.4	-2.1854273504	
F16C3.4	-2.1831877821	+
C04F12.7	-2.1826803744	+
spch-3	-2.1824049931	+
cyb-3	-2.1813258571	+
Y38E10A.17	-2.1810901957	+
F42A9.3	-2.1808865691	
K07H8.7	-2.1789277128	+
spch-1	-2.1781865489	+
B0379.7	-2.1780418624	+
T13F3.8	-2.1770752938	+

B0379.2	-2.1764480937	+
snf-2	-2.1753427168	+
spch-2	-2.1713609007	+
F36H1.3	-2.1707634130	+
aptf-2	-2.1701131925	+
clp-8	-2.1696981396	+
Y71G12B.22	-2.1686691686	
gsp-4	-2.1686296497	+
F54F7.3	-2.1664267973	
K02F6.7	-2.1658048072	+
Y57G11A.2	-2.1645108429	
Y51H7C.9	-2.1642963976	
R10E4.6	-2.1642897000	+
W03B1.9	-2.1628427935	
phy-3	-2.1620474721	+
C45G9.9	-2.1619033913	+
T23F11.2	-2.1618877751	+
T28B8.6	-2.1607116624	
C01G12.9	-2.1600530992	+
Y57G11C.23	-2.1594072162	
F58E6.5	-2.1575299528	+
W04A4.2	-2.1542001051	+
Y43C5B.3	-2.1538011039	
W04E12.4	-2.1518734123	+
K12B6.11	-2.1502826403	
irld-8	-2.1501747290	+
nspb-10	-2.1500406686	
C35B1.4	-2.1497977466	+
F21D9.2	-2.1491867144	
smz-2	-2.1474920267	+
K10D6.3	-2.1438822185	+
ZC250.5	-2.1423167132	
cyp-31A5	-2.1418780203	+
pph-4.2	-2.1408562036	+
C43G2.3	-2.1407263633	+
ZK353.3	-2.1404219087	
clec-209	-2.1379370605	
C02F5.5	-2.1378350827	+
ZK354.6	-2.1363458799	
nep-19	-2.1350785416	
F25H5.8	-2.1343617390	+
R02F2.4	-2.1341662963	+
W09D6.4	-2.1337294810	+
F56A4.2	-2.1320600165	
Y57G11C.52	-2.1319759800	
C27D6.11	-2.1316703320	+
Y53F4B.19	-2.1293269464	
C55C3.4	-2.1283252652	+
F26E4.5	-2.1282380444	+
T16A9.5	-2.1246086250	
snf-5	-2.1243847288	
F54H12.5	-2.1232509843	+
klp-15	-2.1218864631	+
Y53C10A.10	-2.1216665812	+
Y23H5B.12	-2.1213510305	
K01D12.15	-2.1210721409	+

F30A10.13	-2.1200024324	+
Y71G12B.3	-2.1189060923	
F21F3.2	-2.1170722044	+
col-126	-2.1162441444	
clp-6	-2.1161863856	
acdh-5	-2.1154531039	+
K09E4.1	-2.1139269210	+
C17C3.11	-2.1133312502	+
C06A1.3	-2.1132031401	+
R13H9.5	-2.1125997105	+
F40H6.1	-2.1070142142	+
neg-1	-2.1058899408	+
C17H12.12	-2.1030885285	+
F30A10.14	-2.1011576536	+
C27D6.3	-2.0991468303	+
Y116A8C.23	-2.0987865909	
decr-1.1	-2.0971636653	+
T28B8.4	-2.0964891767	
C33C12.4	-2.0932286318	+
K07A3.3	-2.0929401485	+
Y73F8A.20	-2.0923737912	
col-127	-2.0913035623	
C02E7.7	-2.0908239131	+
M28.9	-2.0907950521	+
W03D8.5	-2.0890909060	
Y39E4A.1	-2.0886997040	
C32E8.4	-2.0883279537	+
nep-8	-2.0877752251	+
F55D12.6	-2.0856613981	
cyp-31A2	-2.0847214256	+
htas-1	-2.0829923796	+
K08F4.5	-2.0816084098	+
T05D4.5	-2.0793329199	
F13H8.12	-2.0787168071	+
cav-1	-2.0740851776	+
Y57G11C.14	-2.0736904926	+
K04C2.8	-2.0733132536	
W09C3.8	-2.0726805293	
K06A4.6	-2.0715931517	
K06A4.6	-2.0713135551	
ZK945.7	-2.0711694191	
C31H1.5	-2.0706142655	+
D1086.17	-2.0702020968	+
Y59A8B.12	-2.0694856509	+
C04F12.16	-2.0692720136	
ZK596.2	-2.0685634751	
F11E6.3	-2.0685058842	+
basl-1	-2.0682146768	+
D1022.3	-2.0671858922	+
lab-1	-2.0650412836	+
R07C3.13	-2.0636468535	+
C49F5.6	-2.0620472682	+
H04D03.6	-2.0620286340	+
Y39B6A.30	-2.0586714182	
T04F3.3	-2.0584616987	
sfxn-1.2	-2.0584213207	+

C39B5.5	-2.0576935906	
daf-18	-2.0567207100	+
nspd-9	-2.0564346709	+
Y57G7A.5	-2.0559947502	+
F13E9.5	-2.0536229444	+
col-135	-2.0524306545	+
R10E4.7	-2.0507837628	
W03D8.9	-2.0494727491	
C17G1.2	-2.0477137884	+
Y57G11C.5	-2.0469791160	
Y41C4A.18	-2.0442410574	
F36H12.17	-2.0415305648	+
C36F7.5	-2.0409207392	+
ZK688.10	-2.0388266495	
fis-1	-2.0383320599	
T22B3.3	-2.0372473449	+
W03D8.1	-2.0370138540	
F02E9.3	-2.0368148082	+
Y54E2A.5	-2.0338644934	
C18H7.4	-2.0337966717	+
H12D21.5	-2.0328502185	+
cyp-13A4	-2.0324789308	
F07A5.2	-2.0322486199	+
Y69A2AR.19	-2.0319209469	
gsp-3	-2.0300971992	+
F07G6.10	-2.0296437944	
col-160	-2.0241577573	
pal-1	-2.0238510082	+
nep-24	-2.0236799007	
W06F12.3	-2.0208570122	
B0524.5	-2.0205223272	+
F40F9.3	-2.0200320296	+
Y65B4A.9	-2.0195039170	
ugt-41	-2.0185651992	
M88.3	-2.0176857011	+
F53G12.8	-2.0168293385	+
C16C8.12	-2.0158826922	+
R08C7.8	-2.0150821015	+
F54C1.8	-2.0147030599	
F58F12.2	-2.0142139218	+
ssq-3	-2.0134423367	
ZK945.8	-2.0121562066	
clec-147	-2.0112189437	+
col-162	-2.0109644991	
C30F12.4	-2.0103191220	+
B0207.11	-2.0091346469	
C06E2.5	-2.0069301109	+
R193.2	-2.0066657974	
nspc-7	-2.0059376094	
ify-1	-2.0058745528	+
M110.7	-2.0039262723	+
ssp-36	-2.0016856901	
Y40H4A.2	-2.0014039592	
T28H11.7	-2.0004023834	
F32B6.4	-2.0000783752	+

Note:.-Expression information of candidate genes is from https://wormbase.org.-Statistical significance: $P < 0.01$.
